# Digital Health Behavior Change Technology: Bibliometric and Scoping Review of Two Decades of Research

**DOI:** 10.2196/13311

**Published:** 2019-12-13

**Authors:** Fawad Taj, Michel C A Klein, Aart van Halteren

**Affiliations:** 1 Vrije Universiteit Amsterdam Amsterdam Netherlands; 2 Philips Research Eindhoven Netherlands

**Keywords:** persuasive technology, digital health behavior, behavior change systems, behavior change support systems, bibliometric analysis, scoping review

## Abstract

**Background:**

Research on digital technology to change health behavior has increased enormously in recent decades. Due to the interdisciplinary nature of this topic, knowledge and technologies from different research areas are required. Up to now, it is not clear how the knowledge from those fields is combined in actual applications. A comprehensive analysis that systematically maps and explores the use of knowledge within this emerging interdisciplinary field is required.

**Objective:**

This study aims to provide an overview of the research area around the design and development of digital technologies for health behavior change and to explore trends and patterns.

**Methods:**

A bibliometric analysis is used to provide an overview of the field, and a scoping review is presented to identify the trends and possible gaps. The study is based on the publications related to persuasive technologies and health behavior change in the last 18 years, as indexed by the Web of Science and Scopus (317 and 314 articles, respectively). In the first part, regional and time-based publishing trends; research fields and keyword co-occurrence networks; influential journals; and collaboration network between influential authors, countries, and institutions are examined. In the second part, the behavioral domains, technological means and theoretical foundations are investigated via a scoping review.

**Results:**

The literature reviewed shows a clear and emerging trend after 2001 in technology-based behavior change, which grew exponentially after the introduction of the smartphone around 2009. Authors from the United States, Europe, and Australia have the highest number of publications in the field. The three most active research areas are computer science, public and occupational health, and psychology. The keyword “mhealth” was the dominant term and predominantly used together with the term “physical activity” and “ehealth”. A total of three strong clusters of coauthors have been found. Nearly half of the total reported papers were published in three journals. The United States, the United Kingdom, and the Netherlands have the highest degree of author collaboration and a strong institutional network. Mobile phones were most often used as a technology platform, regardless of the targeted behavioral domain. Physical activity and healthy eating were the most frequently targeted behavioral domains. Most articles did not report about the behavior change techniques that were applied. Among the reported behavior change techniques, goal setting and self-management were the most frequently reported.

**Conclusions:**

Closer cooperation and interaction between behavioral sciences and technological areas is needed, so that theoretical knowledge and new technological advancements are better connected in actual applications. Eventually, this could result in a larger societal impact, an increase of the effectiveness of digital technologies for health behavioral change, and more insight in the relationship between behavioral change strategies and persuasive technologies' effectiveness.

## Introduction

In the past two decades, researchers have spent a lot of effort to understand how digital technology can help people to make a positive change in their behavior. This research is often motivated by the increasing cost of our health care systems and the increasing demand for health care professionals. The field of influencing or changing human behavior through digital technologies started with the term *persuasive technology* (PT) around 1998. Fogg [1] states that persuasion is more than just computer-mediated communication but focuses on human-computer interaction. He defined PT as “how people can be persuaded when interacting with the technology and adjusting itself according to the actions, inputs, and context of persuaded party” [1]. Over time, many terms have emerged to describe technology-based behavior change interventions. For example, Oinas-Kukkonen and Harjumaa [2] define *persuasive systems* as “a computerized software or information system designed to reinforce, change, or shape attitude or behavior or both, without using coercion or deception”. Later, from system perspective, Oinas-Kukkonen [3] defined the term *behavior change support systems* (BCSS): “a sociotechnical information system with psychological and behavioral outcomes designed to form, alter, or reinforce attitudes, behaviors or an act of complying without using coercion or deception”. In this paper, we will use the terms “persuasive technology,” “behavior change support systems,” and “digital health interventions” intermixed.

Often, behavior change support systems are used to support individuals in making lifestyle changes that will lead to better health. When a sufficiently large portion of the population starts making positive changes to health-related behaviors, this will lead to lower utilization of health care and, eventually, to a significant reduction of health care expenditure. Although the potential and societal impact of digital health interventions is highly attractive, so far, its impact on health care utilization and expenditure has been minimal. This is partly because of the limited deployment and implementation, in terms of potential beneficiary users, of effective digital technology for behavior change.

The development of BCSS for health requires an interdisciplinary approach. For example, social psychology provides us a useful theory about the role of people’s personalities, cognitions, and social environment, which system designers could use to better design and develop an effective system. The social science theories provide an accumulated understanding of what human behavior is and the contexts in which they occur, what are the mechanisms of action for change, and what are the ingredients required for change [4]. However, such theories are not often used when designing an intervention [4]. Note that the question *whether an intervention based on theory is more effective* is still under discussion; some reviews have found a positive association [5], whereas others found a negative association [6]. In this study, we tried, among other goals, to explore the adoption theoretical knowledge from related behavioral and psychological sciences, but we did not investigate the effectiveness itself.

In behavioral sciences, 92-item taxonomy of behavior change techniques (BCTs) has been developed to better report about behavior change interventions [7] and support their development. Moreover, some popular models and frameworks exist that provide a systematic procedure for understanding and changing behaviors. For example, Fogg [8] introduced a model called Fogg behavioral model that explains why a person does what he does and also defines functional triads of a persuasive system. Harjumaa extended Fogg’s study and created a conceptual framework called *persuasive system design* (PSD) that can be directly applied for persuasive system development and evaluation [9,10]. Michie et al [11] combined 19 frameworks to propose a new framework called the *behavior change wheel* that comprises a layered structure that explains behavior with 3 components (capability, motivation, and opportunity) and links this to techniques known to change behavior and the concerned policy. There are some more latest models that combines the other well-established models and theories to translate clinical aims into behavioral strategies and endeavor to include the full scope of elements from behavioral principles to technological features [12,13].

To our knowledge, no bibliometric analysis of scientific literature on digital technologies for health behavior change has been published. However, a lot of research has been conducted on different aspects of PT and health domains, for example, the literature about the persuasive design principle in different technology domains [14-17] or systematic reviews about the effectiveness and prevalence of the persuasive systems [18-20]. Only a few studies evaluated the effectiveness of PT for health and well-being; it was hard to establish long-term effectiveness because of the lack of long-term evaluation methods.

Our study is related to the papers mentioned above, but in our study, the main focus is the application domains and the theoretical basis (eg, behavioral theories and BCTs) of actual BCSS. A better understanding of the main application domains, the usage of theories, and the gaps between them will help identify which areas of PT have sufficient evidence that implementation on a larger scale can be justified and which areas still require additional research.

In this study, we first conducted a bibliometric analysis of the literature to answer the questions about the quantitative trends in the literature and the geographical distribution of the researchers. With the help of a co-occurrence network of keywords and research fields, we tried to uncover meaningful insights based on the strength of links between the nodes in the network. We also studied the collaboration between scholars in the field and the collaboration between developers of digital interventions and health behavior change researchers. This is done at the author, institution, and country level.

Second, we have presented a scoping review that aims to critically evaluate the content of the published literature on digital health behavior change systems and answer the following questions: (1) *what are the trends in adopted technologies for different health domains*? and (2) *what are the theoretical foundations (ie, theories from behavioral sciences or systematic frameworks/models for the development of PT) of implemented systems?*

Finally, we concluded with a discussion on the identified pitfalls and possible future directions for research.

The information provided by this review will help analyze who are the relevant stakeholders that have contributed to the growing knowledge of digital health interventions and will help designers make more informed decisions regarding the development and design of PT for healthy behavior change.

## Methods

This section describes the procedures that have been used in the different phases of our study. We discuss the query selection, the database selection, methodologies, eligibility criteria, data items, and the choice of the tools used for different types of analysis.

### Data Collection

We chose to use the Web of Science (WoS) core collection and the Scopus database as basis for the study. The search query aimed to retrieve actually implemented behavior change systems with a technical component. Therefore, the following terms were used: (“persuas*” OR “ehealth” OR “mhealth”) AND (“ICT” OR “prototype” OR “techno*” OR “system”) AND “behavio* change.” The search was limited to the articles publicated during January 2000 and December 2018. The process of merging the collected data is discussed below. The query search resulted in a set of 317 and 325 articles from WoS and Scopus, respectively. Among the 325 items from Scopus, 11 appeared empty or incomplete at further inspection, so 314 were left.

### Study Design

For evaluating the existing scientific output through citations count, keywords, geographical data, authors collaborations, and discipline-wise interactions, the method of bibliometric analysis was used. The basic statistics included yearly publication output, publishing countries, the field of study, citation count, keyword co-occurrences, coauthorships, and collaboration networks between countries and institutions.

Several software packages are available to support a bibliometric analysis, each with different capabilities and limitations. Some of the most popular tools include HistCite [21], CiteSpace [22], BibExcel [23], and Science of Science (Sci2). In this study, the Sci2 tool was used, which is based on the Cyberinfrastructure Shell toolset, for the study of science [24].

In addition to tools for bibliometric analysis, we also used network visualization and analysis tools for the co-occurrence networks of keywords and research fields and the collaboration networks between countries and institutions. We used Gephi and VOSviewer that use a 3-dimensional render engine to render illustrations of large networks [25,26]. For the network analyses, the 394 extracted articles and their references were converted into graphs. For example, for the coauthor network, 394 articles resulted in 1777 nodes, where each node represented an author, and 5583 edges, where each edge represented coauthored studies.

A scoping review is a useful methodology to determine the coverage of a body of literature on a given topic and identify and analyze knowledge gaps [27]. A number of research questions in the introduction section were defined that will help investigate the use of behavior change theories and BCTs in actual BCSS.

Owing to the broad scope of the review, any conventional systematic review or meta-analysis framework was not followed strictly. However, the Preferred Reporting Items for Systematic Reviews and Meta-Analyses for Scoping Reviews guidelines (PRISMA-Scr) were applied [28] for our scoping review.

### Eligibility Criteria

To be considered for review, the following inclusion criteria were formulated: (1) publication in a peer-reviewed academic journal; (2) publication in the English language; (3) the research should have the primary purpose of changing behavior, either increasing or decreasing the behavior or stopping the behavior altogether; and (4) the article should discuss a technological solution that is used for behavior change.

### Categorization

In this section, the key categories used in this study to classify the different interventions described in our dataset have been defined. The first category, *technology*, refers to the type of computer-based tool that is used to change a behavior, that is, a digital device, hardware, or a software solution. The *target domain* describes the behaviors that are targeted for change—for example, diet, sedentary behavior, mental health, or physical activity. The term *behavior change theory/model* is used to describe the theories and models about behavior change from behavioral sciences that are used as the basis for the persuasive systems. The category *development frameworks/models* covers a slightly different aspect; it describes the frameworks and models that are used to guide the design and development of the intervention. To further clarify the distinction between the two categories discussed above, the behavior change theory/model is used as the key behavior change principle for approaching the problem. For example, in a study by Lyons et al [29], the authors approached narrative transportation theory as a basis for increasing autonomous motivation about health behavior. In contrast, elements of the development frameworks category provide a systematic approach to select the behavioral principles and technological features during the design and development of the system. Finally, the category *BCT* describes the active ingredients of an intervention, for example, goal setting, reminder, and education. For this category, the taxonomy found in the study by Michie et al [7] was used.

Table 1 provides an overview of the categories and some examples of values. The categorization of the systems has been performed by the first author and finalized with the approval of other authors.

**Table 1:** Classification and coding scheme.

Category	Possible values
Technology	Web, mobile app, computer applications, mobile game, SMS, pedometers, virtual agent, and interactive voice response
Target domain	Physical activity, healthy eating, smoking cessation, carbon emission, and energy consumption
Theories/model employed for behavior change	Transtheoretical model, motivational interviewing, health belief model, and social cognitive theory
Development frameworks/model	Persuasive system design, behavior intervention technology, intervention mapping, and behavior change wheels
Behavior change techniques	Self-monitoring, motivation, goal setting, reward, punishment, and knowledge

## Results

### Data Source and Selection

We applied our search query on 2 major literature databases, that is, WoS and Scopus. The WoS and Scopus databases returned 317 and 314 articles, respectively. There was an overlap of 179 articles between both datasets; thus, we identified 452 unique articles.

To perform our *bibliometric analysis*, we ideally would have merged the data from WoS with the data from Scopus. However, there are some technical constraints when performing a bibliometric analysis on different datasets: first, each database uses different sources for indexing the articles and, second, each database starts to index different journals at a different moment in time. For example, WoS started indexing the *Journal of Medical Internet Research* and its sister journal articles in 2015, whereas Scopus started in 2018 and is still indexing only a few of the journals published by JMIR Publications. Another issue is that the citation count for each article is different in the different databases, apparently, because different sources are used.

Owing to its quality and completeness of data, we decided to base our bibliometric analyses on data extracted from the WoS database [30,31]. To include as many relevant papers as possible, a manual search was performed in the WoS database for the 135 (314 minus 179 overlap) Scopus results that were not returned by the search query in WoS. Of those 135 articles, 77 were found in the WoS database, and 58 were not found. This resulted in a dataset of 394 items (317 as results to a query and 77 manually added items) for the bibliometric analysis.

**Figure 1:**
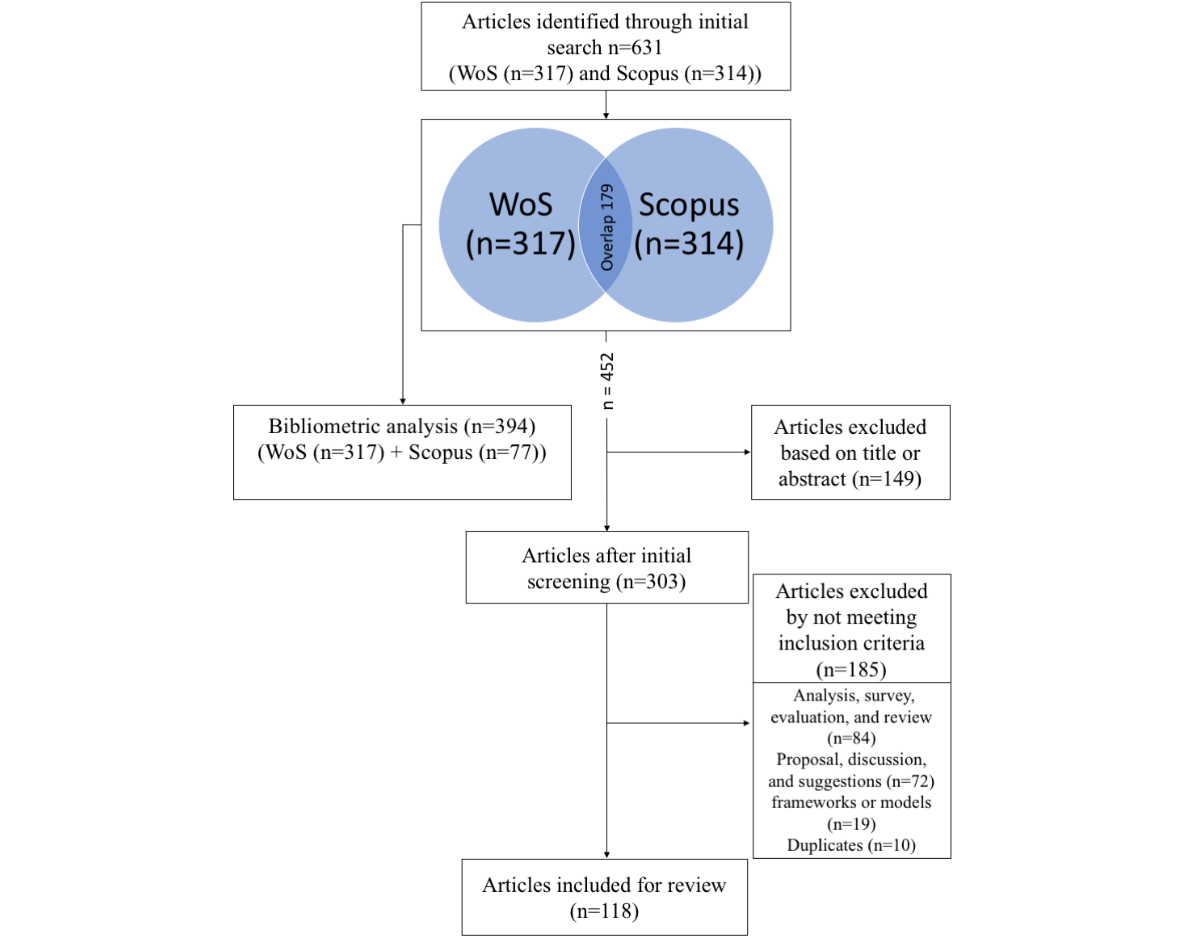
Flowchart of study selection (n=the actual number of publications). WoS: Web of Science.

Our *scoping review* started from the full set of 452 unique articles. A 2-phase screening was performed to determine their relevance for our scoping review. The first author initially screened the articles by reading the title and abstract and removing those that clearly did not match the inclusion criteria. As a result, 149 articles were excluded. The remaining 303 articles were thoroughly studied by the main author and compared with the inclusion criteria, which led to the exclusion of another 175 articles. The excluded articles were characterized in 3 main categories through group discussion. Furthermore, 10 articles were found as duplicates, as they were discussing the same intervention. Eventually, 118 articles were considered for the scoping review. The detailed flow of the selection process is presented in Figure 1.

### Bibliometric Analysis

#### Growth of the Literature and Distribution Over Countries

**Figure 2:**
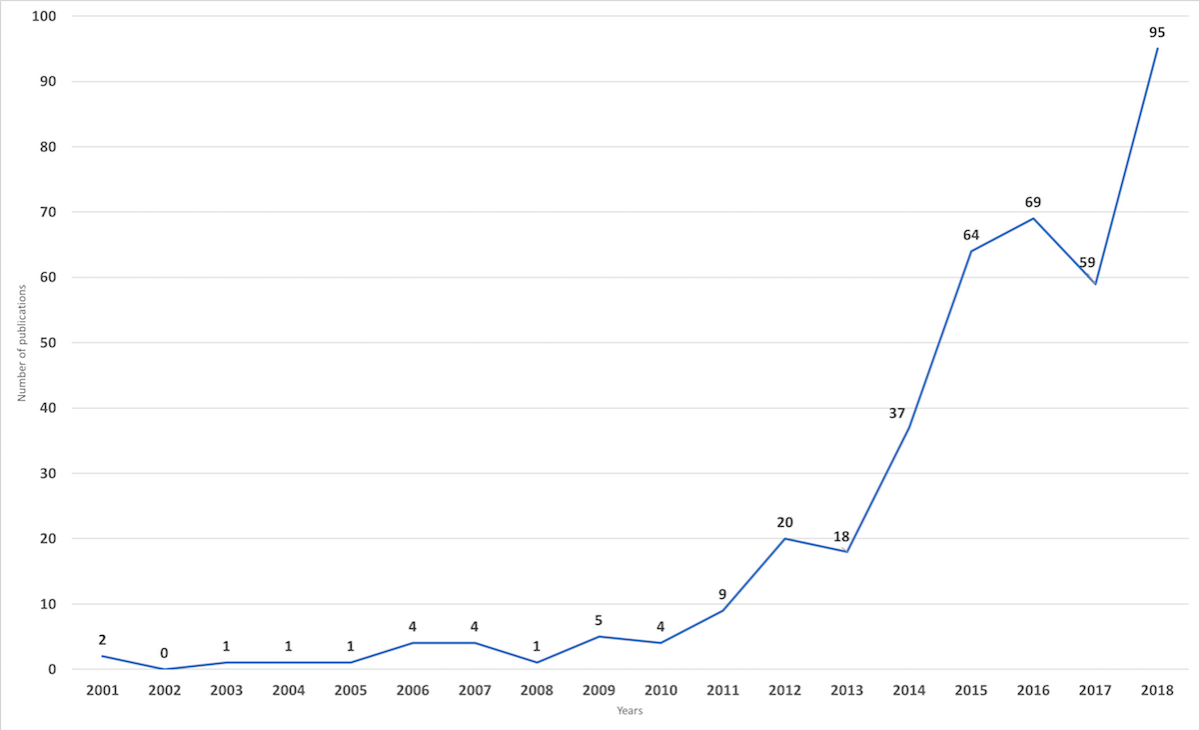
Publication trend from 2000 to 2018.

When looking at the number of publications in our selection, we can observe that it started in 2001, with 1 or 2 publications yearly, but rapidly increased since 2009 to 2010, as can be seen in Figure 2.

Table 2 shows the top 10 regions where publications originated. The United States is leading with 46.5% (183/394 records) of all contributions, followed by England and the Netherlands with 14.5% (57/394 records) and 8.4% (33/394 records), respectively. Furthermore, Australia and Canada are fourth and fifth in the ranking with 7.6% (30/394 records) and 5.8% (23/394 records), respectively.

**Table 2:** Persuasive technology for behavior change, scholarly papers by region.

Country	Publications, n (%)
United States	183 (46)
England	57 (14)
The Netherlands	33 (8)
Australia	30 (7)
Canada	23 (6)
New Zealand	19 (5)
Finland	17 (4)
Italy	16 (4)
Belgium	13 (3)
Switzerland	11 (2)

#### Interdisciplinary Collaboration Network

**Figure 3:**
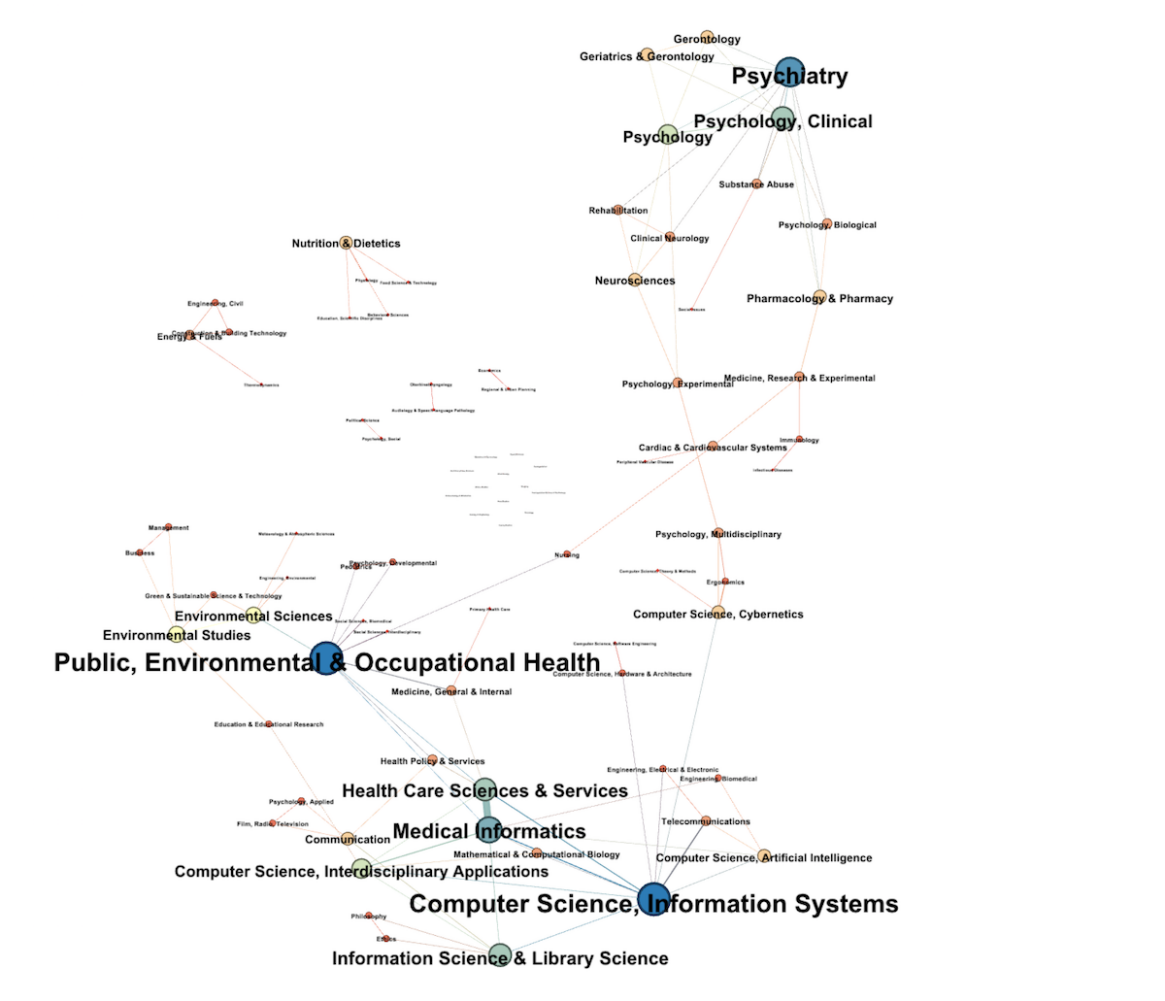
Disciplines involved in persuasive technology and health behavior change.

From the *research field* column in the WoS database, a co-occurrence network of research areas has been created. The weights of the nodes in the graph were determined by the number of publications in each given category. The visualization of this network (see Figure 3) shows 3 clear clusters. The first cluster is the area of computer science and related fields, the second cluster is public health care and occupational health, and the third major cluster is psychology and subfields.

#### Citations

Tables 3 and 4 show the list of the 5 most cited articles, either globally or locally. The global citation count is based on the full WoS citation count, whereas the local citation count is the number of citations of an article within the network.

**Table 3:** Top 5 of the globally most citied articles.

Title	Reference	Global citation count
A behavior change model for internet interventions	[32]	235
New directions in electronic health communication: opportunities and challenges	[33]	205
Behavior change techniques implemented in electronic lifestyle activity monitors: a systematic content analysis	[34]	162
Virtual self-modeling: the effects of vicarious reinforcement and identification on exercise behaviors	[35]	151
Online interventions for social marketing health behavior change campaigns: meta-analysis of psychological architectures and adherence factors	[36]	139

**Table 4:** Top 5 of the most citied articles within the network (locally).

Title	Reference	Local citation count
Health behavior models in the age of mobile interventions: are our theories up to the task?	[37]	45
Behavior change interventions delivered by mobile telephone short message service	[38]	44
Text messaging as a tool for behavior change in disease prevention and management	[39]	43
The theory of planned behavior	[40]	41
Persuasive technology: using computers to change what we think and do	[41]	41

#### Co-occurrence of Keywords

**Figure 4:**
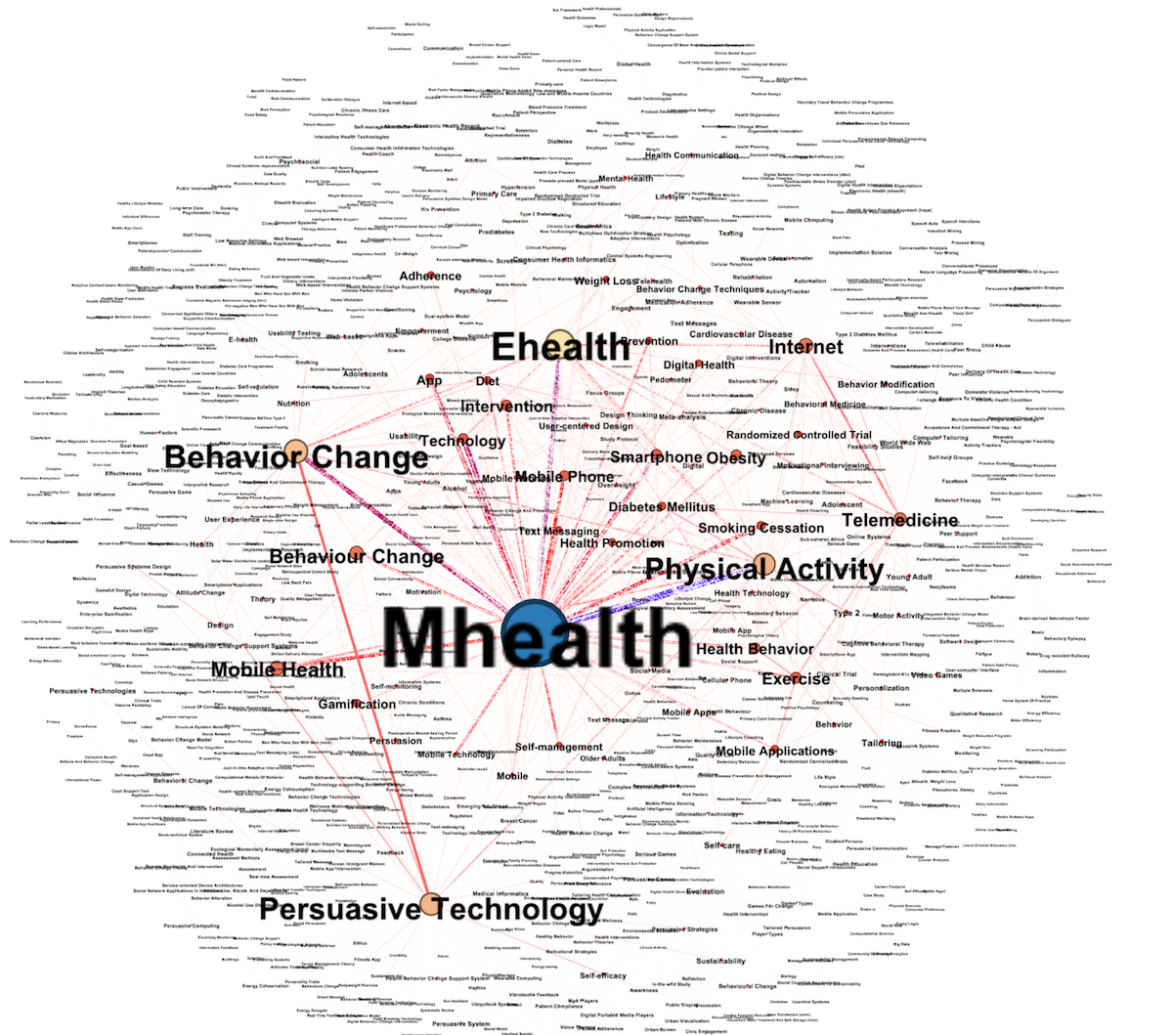
The co-occurrence network of author keywords.

keywords are assumed to compose an adequate description of the content of a research article. The co-occurrence of keywords could provide an interesting structure of the research field, as it reveals the semantic relations in the scientific literature. The most frequently used keywords are “mhealth,” “physical activity,” “ehealth,” “persuasive technology,” “smart phone,” and “behavior change” (see Figure 4). The size of the node and label reflects the co-occurrence count of a certain word. The higher the count, the larger the size of node and label.

#### Coauthorship Network

**Figure 5:**
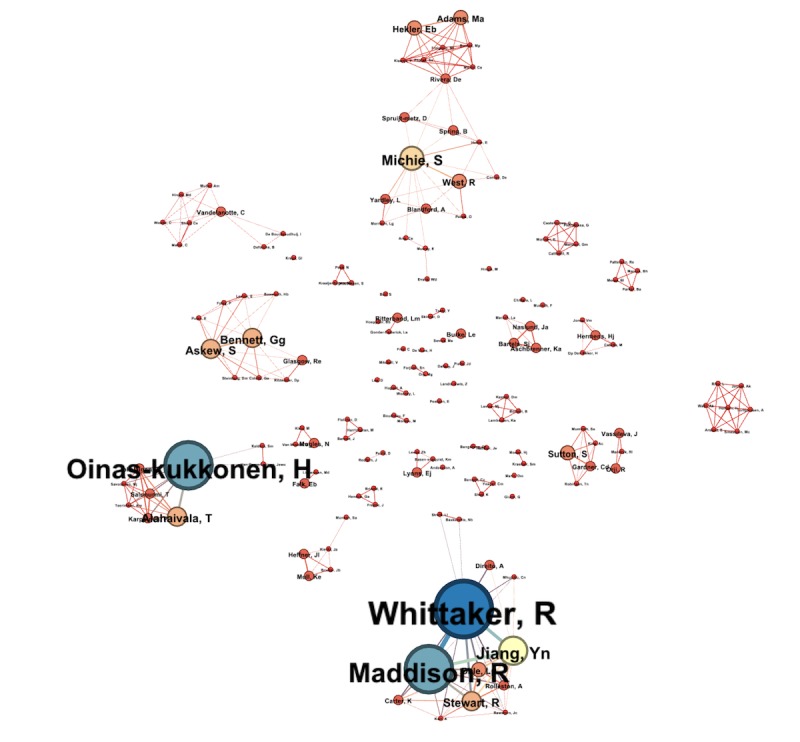
Coauthor graph.

A coauthorship network was extracted using the Sci2 tool. Each node represented an author, and a connection between 2 nodes represented a coauthorship. A total of 1777 authors were identified with 5583 connections. For better visualization, only authors who had at least two publications together were considered. Node sizes were based on the number of articles coauthored with other authors. There were 3 strong clusters among the network that were strongly intraconnected but not interconnected (see Figure 5). In Figure 5, the highest coauthorship count (13 times) within the network was by R Whittaker. Her major coauthorship is with colleagues R Maddison and Y Jiang at the National Institute for Health Innovation, University of Auckland, Auckland, New Zealand. The other cluster is led by Oinas-Kukkonen, with a coauthorship count of 11 and most coauthorships with his colleague, T Alahäivälä, at the University of Oulu, Faculty of Information Technology and Electrical Engineering, Oulu Advanced Research on Service and Information Systems, Oulu, Finland. The last cluster is led by S Michie (Research Department of Clinical, Educational, and Health Psychology, University College London or UCL, London, United Kingdom), with a coauthorship count of 6 and, mostly, with R West (Health Behavior Research Center, UCL Epidemiology and Public Health, London, United Kingdom) and EB Hekler (School of Nutrition and Health Promotion, Arizona State University, Phoenix, Arizona, United States).

#### Most Important Journals

The articles under review were published in 147 different journals. Table 5 shows the distribution of published articles in the top 6 journals. The leading journals are all related to JMIR Publications. The most often used subjournal is *JMIR mHealth and uHealth* with 45 publications out of 394 (11.4%). The main journal, *Journal of Medical Internet Research*, and the subjournal, *JMIR Research Protocols*, with 34 (8.6%) and 17 (4.3%) are in second and third places, respectively.

**Table 5:** List of top journal distribution.

Journal title	Publications (n)
*JMIR mHealth and uHealth*	45
*Journal of Medical Internet Research*	34
*JMIR Research Protocols*	17
*BioMed Central Public Health*	11
*Personal and Ubiquitous Computing*	10
*International Journal of Medical Informatics*	9

#### International Collaboration

**Figure 6:**
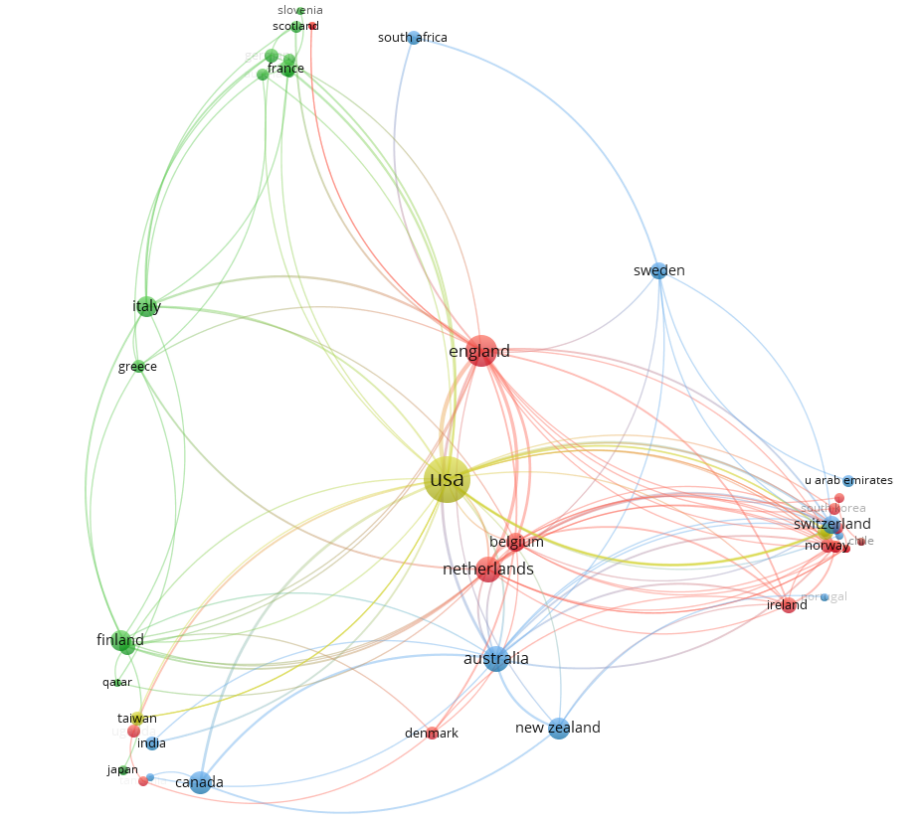
Countries collaboration graph.

To investigate the collaboration between countries and organizations/universities, the geographical distribution of the research was analyzed. Coauthorship networks between countries and organizations/universities were extracted. Figure 6 shows the network of 52 productive countries in 8 clusters; each cluster is represented by different colors. The size of the node represents the number of articles originating from a certain country and the thickness of the connection indicates the number of collaborations between the 2 countries. The largest cluster is the red one with around 18 nodes, led by England. The second biggest cluster is the blue one (12 nodes); the major country in this group is Australia.

**Figure 7:**
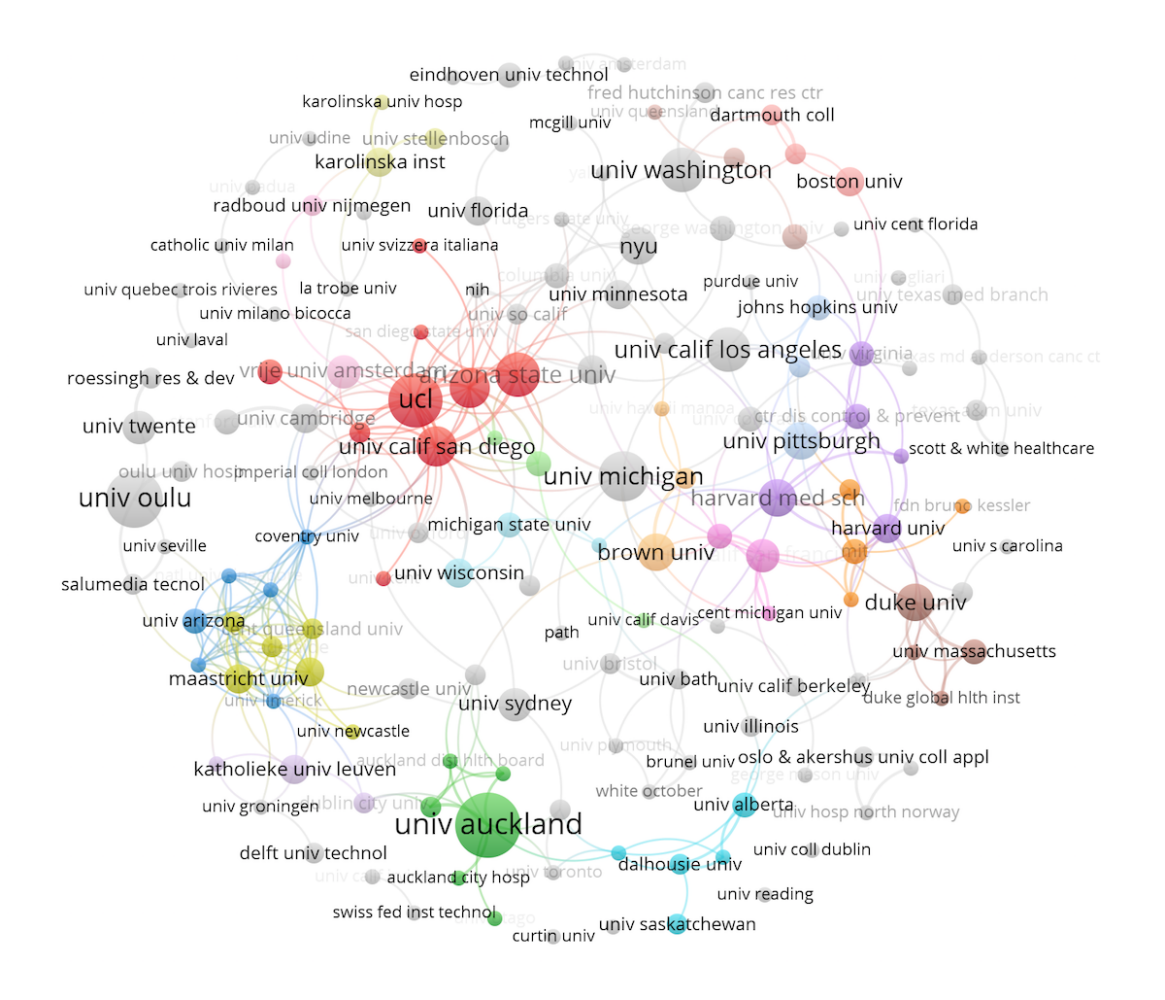
Organization/institution collaboration graph. Coll: college; Hosp: hospital; Inst: Institute; NYU: New York University; Technol: technology; UCL: University College London; and Univ: University.

Figure 7 shows the productiveness of institutions and their collaboration with other institutions. There were about 511 organizations identified, but we only considered the organizations that had at least two or more articles published. This filter left us with 90 institutions, which were divided into 10 clusters, each depicted with a different color. The largest and most productive cluster is the red one with 20 nodes. Most institutions in the cluster are from the United Kingdom and the United States, with UCL and Arizona State University as the leading ones. The second largest cluster is the blue one, with 12 nodes and University of Michigan and University of California as leading institutions. The size of nodes reflects the number of documents, and the thickness of the edge reflects the strength of collaboration.

### Scoping Review

The second objective of this study was a scoping review. The contents of the 118 articles that resulted from our selection process were thoroughly studied. This revealed some interesting insights and trends. The findings about the trends regarding technological choices and the theoretical basis of digital health interventions are presented in the following sections.

#### Technology Platforms Used

Table 6 summarizes the major technological platforms used for persuasive systems for behavior change. Apps on a mobile phone are the most frequently employed platform with 52.5% (62/118) of the studied systems, followed by SMS and the Web with 21.1% (25) and 19.4% (23), respectively. Less frequently adopted platforms are wearable sensors 16.1% (19), games 6% (8), desktop apps 3% (4), and social media 2% (3). Finally, 9.3% (11) use yet another platform. A complete list of technology usage with references is provided in Multimedia Appendix 1.

**Table 6:** Frequency of different technological platform used.

Digital technology	Usage count, n (%)
Mobile apps	62 (52)
SMS	25 (21)
Web	23 (19)
Wearable sensors	19 (16)
Others	11 (9)
Game	8 (6)
Desktop apps	4 (3)
Social media	3 (2)

#### Targeted (Health) Domains

The most often targeted behaviors are health-related behaviors. The top 8 is formed by physical activity, healthy eating, diabetes management, smoking cessation, weight control, AIDS/sexual behavior, cardiovascular diseases, and alcohol consumption. Physical activity makes up 28.8% (34/118) of all the reviewed studies, followed by healthy eating and diabetes with a total of 18.6% (22) and 11% (13), respectively (see Table 7). There are also some interventions targeting multiple health domains, for example, with physical activity for both diabetes control and cardiovascular diseases.

**Table 7:** Different targeted behavioral domains.

Targeted behavior	Count, n (%)
Physical activity	34 (28)
Healthy eating	22 (18)
Diabetes management	13 (11)
Smoking cessation	10 (8)
Weight control	10 (8)
AIDS or sexual behavior	6 (5)
Cardiovascular disease	5 (4)
Carbon dioxide emission	5 (4)
Energy saving	4 (3)
Alcohol consumption, medical adherence, lower back pain, mental illnesses	3 (2)
Overdose prediction, mammography adherence, asthma control, sedentary behavior, knee osteoarthritis, waste management, educational behavior	2 (1)
Psychotropic, multiple sclerosis, sleeping behavior, screen time	1 (0.8)

#### Behavior Change Theories

Most of the analyzed articles seem not to be based on proper theories. Only 33% (59/118) of articles reported at least one or more theories among the 21 theories identified during out review for designing the system. The social cognitive theory (SCT), transtheoretical model, self-determination theory, and motivational interviewing are most frequently reported theories. Table 8 shows the top 5 of most frequently used theories. The complete pie chart showing the usage percentages of all reported theories is provided in Multimedia Appendix 1.

**Table 8:** Percentage of reported theories (N=59).

Theory	Number reported, n (%)
Social cognitive theory	17 (29)
Transtheoretical model	6 (10)
Self-determination theory	4 (7)
Motivational interviewing	4 (7)
Theory of planned behavior	3 (5)

#### Framework/Model Adopted

Another important finding is about the usage of development frameworks and models. Such frameworks provide guidance on the development of a persuasive system, by suggesting a *coordinated set of activities* to help translate theory into practice [11]. Similar to the result of theories usage, we also see that the usage percentages are quite low; only 47 articles used any framework or model. We found a total of 15 frameworks, but they appeared to be rarely followed (see Table 9). However, it is also found that *gamification* is increasingly used as a paradigm for developing persuasive systems. Gamification is generally understood as the integration of specific features (eg, points, leaderboards, levels, competitions, rewards, and achievements) into the wider context of pursuing a goal [42]. These features are not only used in designing game-based interventions but also equally popular in other technology-based interventions. Among the list of 15 frameworks and models, PSD is a popular development framework with 20% (9/47) usage. The complete pie chart of reported frameworks/models is provided in Multimedia Appendix 1.

**Table 9:** Usage of different development framework/models (N=47).

Framework/model	Usage percentage, n (%)
Persuasive system design	9 (20)
Gamification	8 (17)
User-centered design	4 (9)
Intervention mapping	4 (9)
BJ Fogg persuasive principles and model	4 (9)
Theoretical domains framework	2 (4)

#### Behavior Change Technique Employed

**Figure 8:**
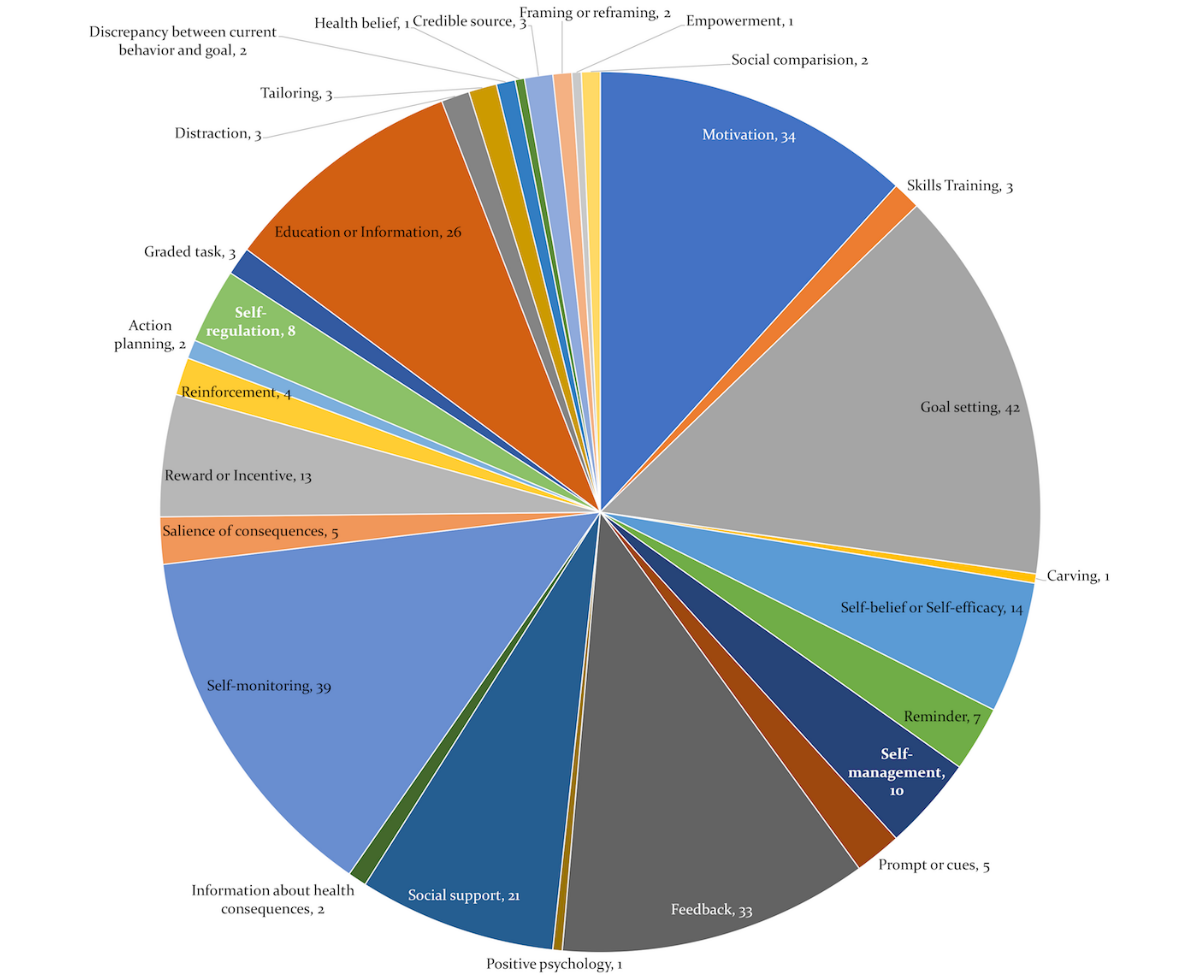
Frequency of different behavior change techniques adopted.

There are many different BCTs that can be used to induce behavior change. Goal setting is the most frequently employed strategy with a total of 42 studies, followed by self-monitoring and motivation, by 39 and 34 times, respectively. Feedback is used for 33 times (see Figure 8).

It needs to be mentioned that there are no standard guidelines for reporting active components of the interventions, and people used different synonyms to report similar techniques [43]. The recently developed extensively agreed taxonomy of techniques used in behavior change interventions [7] is obviously not reported in papers before 2013 but also in newer papers, it is not often used (maybe partly because it is not widely known [44]). In our review, only 8 articles were identified that used this taxonomy for reporting the BCT adopted in the intervention.

It is relevant to mention that there is some overlap between the psychological constructs and BCTs [43]. Sometimes, articles do not explicitly mention the mechanism of change but do mention the construct they targeted. For example, a study [45] targeted the psychological construct *motivation* through text messaging but did not explicitly mention the type of motivation and what the mechanism of change was. In this part of our analysis, we focused on explicit descriptions of BCTs as mechanism of change. A list of BCTs with references is provided in Multimedia Appendix 1.

#### Relating Targeted Behavior With Technologies

**Figure 9:**
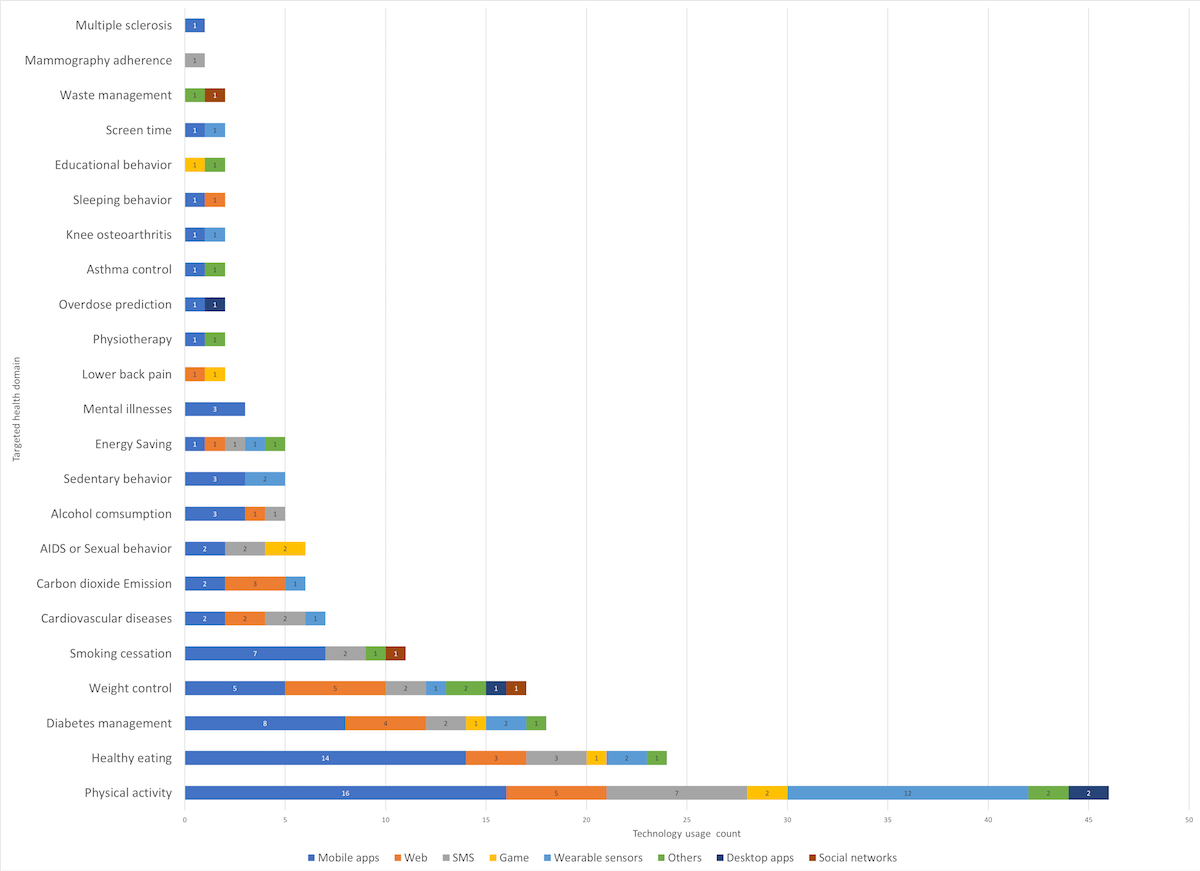
Bar graph representing the different targeted health domains using different technological platforms.

After analyzing the usage of BCTs, theories, and technologies previously, we now analyze a combination of them. First, we compare the targeted behavior with the technological platforms that are used. We found that for increasing physical activity, almost all technological platforms were used, whereas for healthy eating, mobile apps were used in more than 80% of the cases (see Figure 9).

#### Relating Targeted Behavior With Behavior Change Techniques

**Figure 10:**
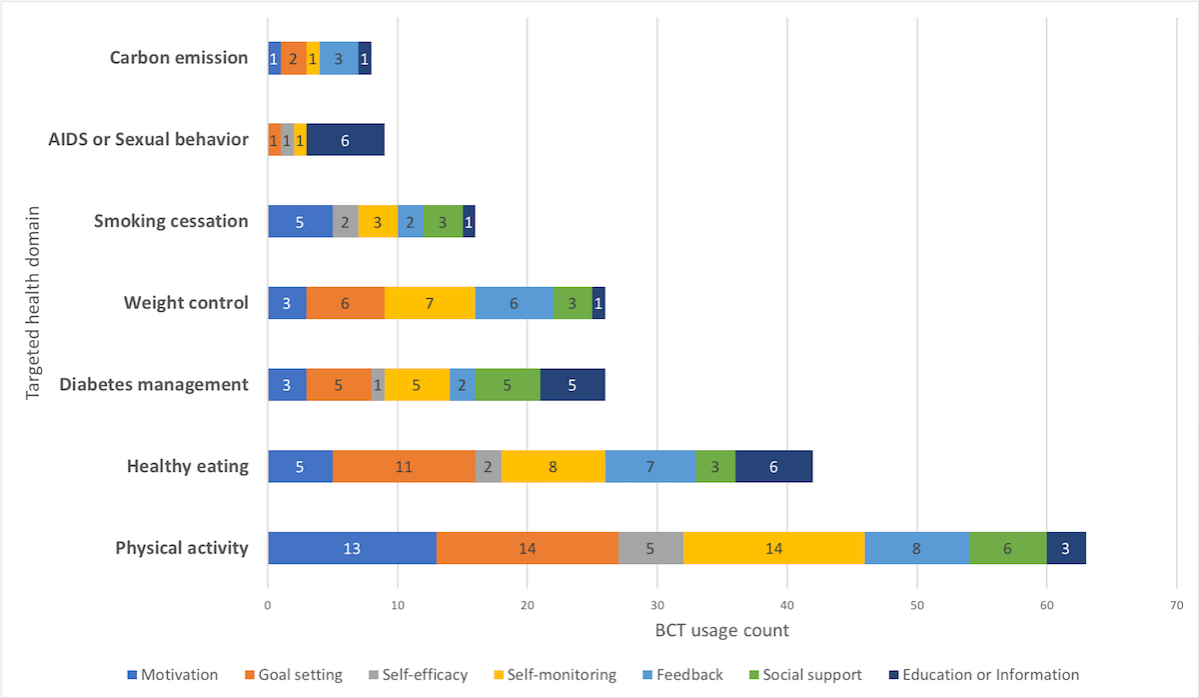
Bar graph represents the different target behavior using different behavior change techniques.

Change in physical activity was targeted by a number of different BCTs, mostly by goal setting, self-monitoring, and motivation. Healthy eating was mostly targeted by self-monitoring, goal setting, and feedback (see Figure 10). From the findings, it seems quite hard to make any one-to-one link between behavior and BCT. The choices of BCTs for each intervention and system vary significantly, and each designer decides on his own accord.

#### Relating Behavior Change Techniques With Technologies

**Figure 11:**
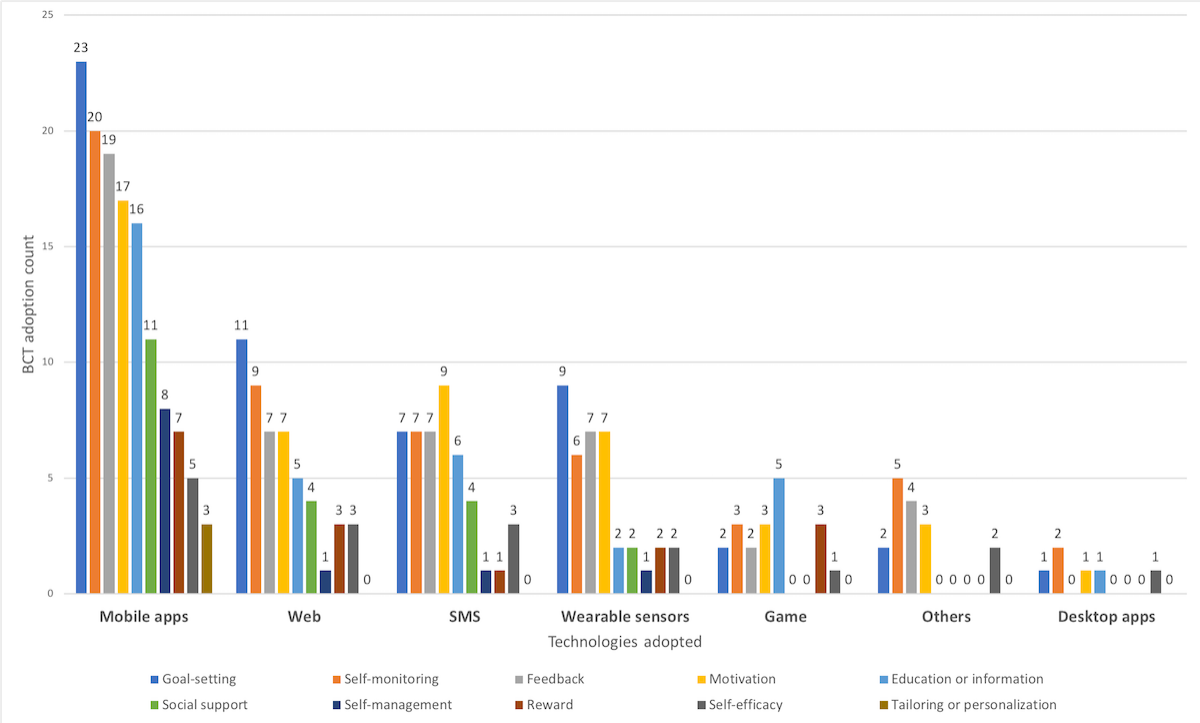
Frequency of different behavior change techniques per technological platform.

Some techniques were more frequently applied in one technological platform than in another. In mobile apps, the most frequent strategies are goal setting (22 times), self-monitoring (20 times), and feedback (17 times; see Figure 11). Almost all the major BCTs are used on the mobile platform, probably because of its flexibility and accessibility. When using game, the most frequently used BCTs are education and reward, as those are important features of gamification. Goal setting is almost evenly used in all technological platforms.

## Discussion

This study presents a comprehensive review of digital technologies and health behavior change. The review comprises 2 parts. First, a thorough bibliometric analysis has been conducted to present the scholarly networks and the global research trends. The bibliometric analysis identifies influential articles, authors, and collaboration networks among different stakeholders and shows where interdisciplinary collaboration is already strong and where further collaboration could strengthen the field. The bibliometric analysis is followed by a scoping review to map the collected literature and answer questions about the theoretical grounding of digital behavior change interventions and the use of technological platforms and targeted domains.

### Bibliometric Analysis

The field of PT is still quite young, and the literature regarding persuasive technologies for health and well-being started to appear in early 2000 [46]. The literature has grown exponentially after 2009/2010, which seems to be because of the introduction of smartphones in this era. Given the development around ubiquitous technologies such as sensors and networks, it is reasonable to expect that the growth of the literature will continue. As the field begins to mature, we see the number of publications increasing and researchers from different fields (eg, psychology and behavior sciences) contributing to the field. The geographic distribution of the publications shows that the United States plays a leading role, with a decent contribution from Western European countries and Australia. Other regions such as Asia and Africa almost have no contributions. To be able to include different behaviors and cultures, it would be useful if the collaboration networks are extended to these regions.

Given the fact that technology for behavior change requires expertise from different scientific areas, we expected quite some collaborations between technological and behavioral scientists. This study shows that interdisciplinary collaboration was not as widespread as expected. As human-centered disciplines such as psychology and other behavior sciences are quite mature and can provide essential knowledge about human behavior, technological researches cannot develop effective digital behavior change interventions without their contribution. Similarly, behavioral scientists require knowledge and insights from technological areas to apply their knowledge with modern means.

The keyword network illustrates the most important knowledge structures and thematic evolution in the field of digital behavior change systems. The keyword “mhealth” was strongly connected with the word “physical activity” and “ehealth.” This finding was not so different from the findings of our scoping review. The author collaboration network can be useful for expanding the collaboration network. The network shows a very strong intracollaboration among 3 groups of authors, where one is specifically working on behavior change, another on mobile health interventions, and the last one on persuasive system or BCSS. There is an opportunity to increase the intergroup collaboration, which could add valuable knowledge to the field. For example, with the help of remote sensors and Internet of Things (IoT) devices, health practitioners can monitor and tune systems for better adherence for their target group.

### Scoping Review

The translation of theories and theoretical frameworks/models into practice is essential for the development of any intervention. Davis et al [4] already concluded that only a few theories were used frequently. We also identified that the theoretical grounding for most of the systems was weak or, at best, not well described. An often-discussed reason for ignoring the majority of the theories is that intervention developers usually choose the most used theories and consider them as an easy and good choice. Another possible explanation is that the PT designer often lacks the skill to translate the theoretical determinants into technology design artifacts that are as effective as originally intended [4]. Conversely, a psychologist could study how digital technology enables new ways to personalize interventions and deliver just-in-time behavior change, with the potential to develop behavioral theories specific to the digital age.

The creation of a taxonomy of BCTs has been an important development in behavioral science, which is visible in the topmost cited articles in this review. However, these BCTs are still underreported in publications that describe persuasive systems [18]. Drawing on [47], the systematic review on mobile apps found that only 10 out of 93 BCTs were mentioned (mean of 2.42 BCTs were present in each app). Our finding in this review is not so different, only 6 articles explicitly used Michie coded taxonomy of BCTs. The most recent addition at the theoretical level is the link between the mechanism of actions and BCTs; this will probably pace up the development and evaluation of effective behavioral change interventions [48]. Owing to nonstandardized reporting, it is very hard to establish any relationship of effectiveness between 1 strategy and the success of persuasive systems. The result shows that each behavior domain is targeted with several techniques and strategies. Therefore, it is quite hard to determine the effectiveness of a certain technique or theory and the recommended process for design and evaluation of a behavior change support system.

### Identified Pitfalls and Future Studies

On the basis our study, we can formulate a number of suggestions for future directions of the research in this domain.

Our study found a large gap in the process of designing digital technologies for health behavior change. They are, usually, weak in their theoretical grounding, and the papers describing them do not clearly report the different components, for example, persuasive strategies, theories, and BCTs. The main reason for this is the lack of design guidelines for these components. For better utilization and reporting of behavioral theories, the development frameworks also need to be updated to the most recent technological advances, for example, the IoT, that is, technologies capable of collecting a large amount of data, such as sensors and mobiles. Furthermore, computational models based on different theories can be designed that could be used by digital intervention developers [13].

Moreover, the relation between BCTs and behavior change theories/mechanisms requires more elaboration. For example, both for the SCT and theory of planned behavior, the use of self-efficacy construct is important. Self-efficacy is the strongest predictor of intention [49]. Furthermore, the correlations between determinants at different ecological levels for different behaviors need to be established. For example, the sitting time in cars has already been associated with urban design [50]. This requires more cross-sectional studies and controlled trials. These studies could help system developers make early decisions about the approach and techniques to follow for their digital interventions.

A clear framework or mechanism for reporting the components of health behavior change systems will not only advance the evaluation and its research design (eg, assess engagement, acceptability, and effectiveness) but also could enhance the costly process of development.

### Limitations

There are 2 limitations to our study worth mentioning. First, owing to technical reasons, we only considered the WoS database for bibliometric analysis; 58 papers that were relevant according to Scopus were not included in our analysis. A second limitation is a possible subjectivity in our scoping review. The categorizing of the reviewed papers has been done in a thorough manner but might still be influenced by subjective interpretations. Unfortunately, this problem cannot be avoided in this type of studies.

## Appendix

Multimedia Appendix 1 Overview over behavior change theories, frameworks, models and techniques used in the included persuasive technology of health.

## Abbreviations

BCSS: behavior change support system
BCT: behavior change technique
ISI: Institute for Scientific Information
IoT: Internet of Things
PSD: persuasive system design
PT: persuasive technology
Sci2: Science of Science
SCT: social cognitive theory
UCL: University College London
WoS: Web of Science
