# Supplementary material for: Digital Health Behavior Change Technology: Bibliometric and Scoping Review of Two Decades of Research
Source: JMIR Mhealth Uhealth. 2019 Dec 13;7(12):e13311. doi: 10.2196/13311 (PMC6935048; doi:10.2196/13311)
Supplement: Multimedia Appendix 1 [file mhealth_v7i12e13311_app1.pdf]

Multimedia Appendix 1 Overview over behavior change theories, frameworks, models and techniques used in the included PT of health.

Figure 1. The frequencies of the adopted theories. EMA: Ecological Momentary Assessment; MI: Motivational Interviewing theory; TTM: Transtheoretical Model; I.B.C.M: Integrated Behavior change model; TPB: Theory of Planned behavior; SDT: self-determination Theory; SCT: Social cognitive theory; FRAMES: Feedback, Responsibility, Advice, Menu of options, Empathy, and Self-efficacy; GST: Goal-setting Theory; HF: Habit Formation; TT: Temporal Tensions; NRT: Narrative transportation theory; CBT: Cognitive behavioral theory; HBM: Health Belief Model; SRT: Self-Determination Theory; WMT: Wellness Motivation theory; TRA: Theory of reasoned action; KAB: Knowledge-attitude behavior.

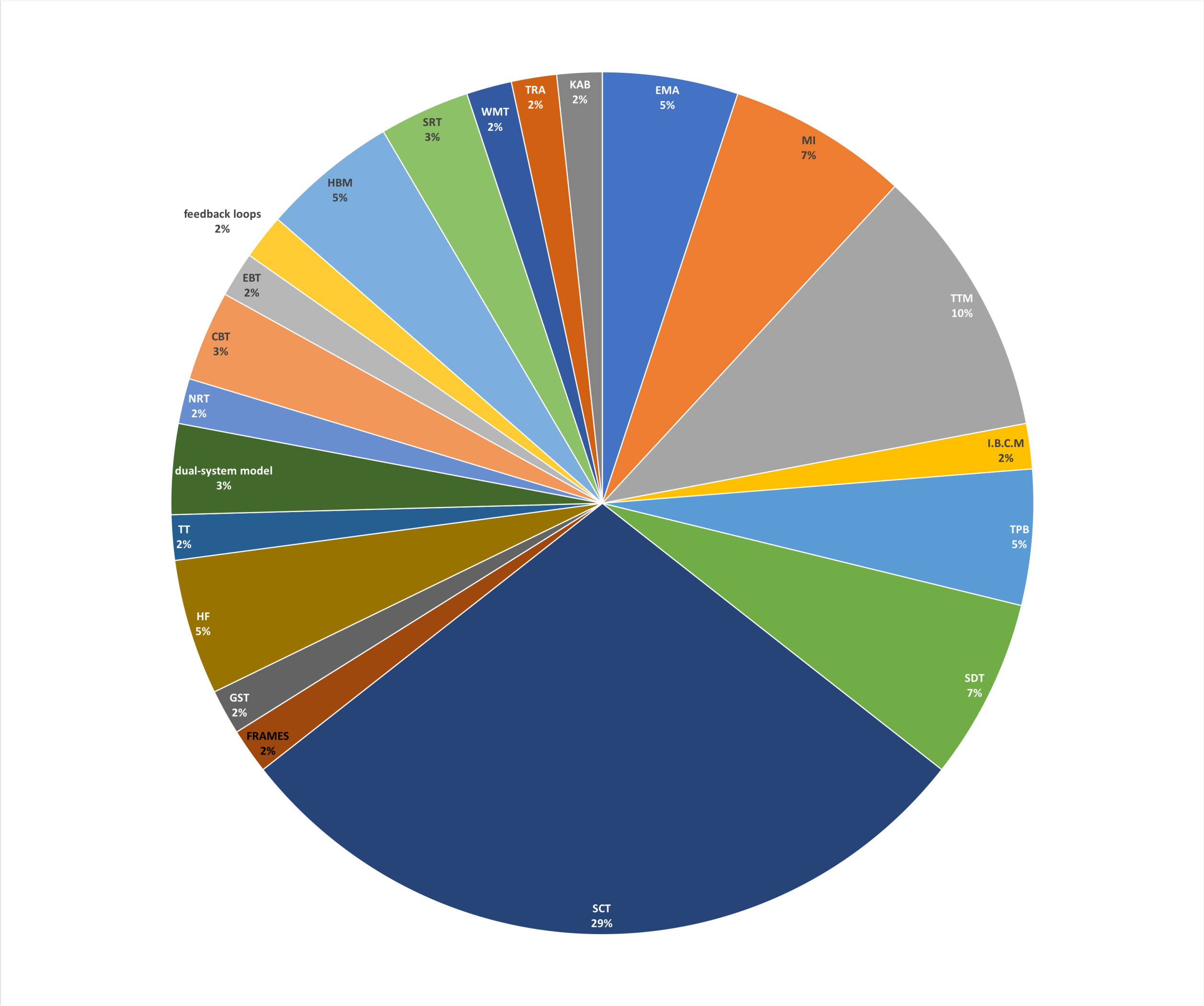

Figure 2. The frequencies of the development framework/models adopted. PSD: persuasive system design; BCW: Behavior Change Wheel; BITF: Behavior Intervention technology framework; IM: Intervention mapping; TDF: Theoretical Domains framework; STAR: Spiral Technology Action Research (STAR) model; UCD: User centered design; RE-AIM Reach, Effectiveness or efficacy, Adoption, Implementation, Maintenance; IDEAS: Integrate, Design, Assess, and Share; FBM: Fogg behavior model.

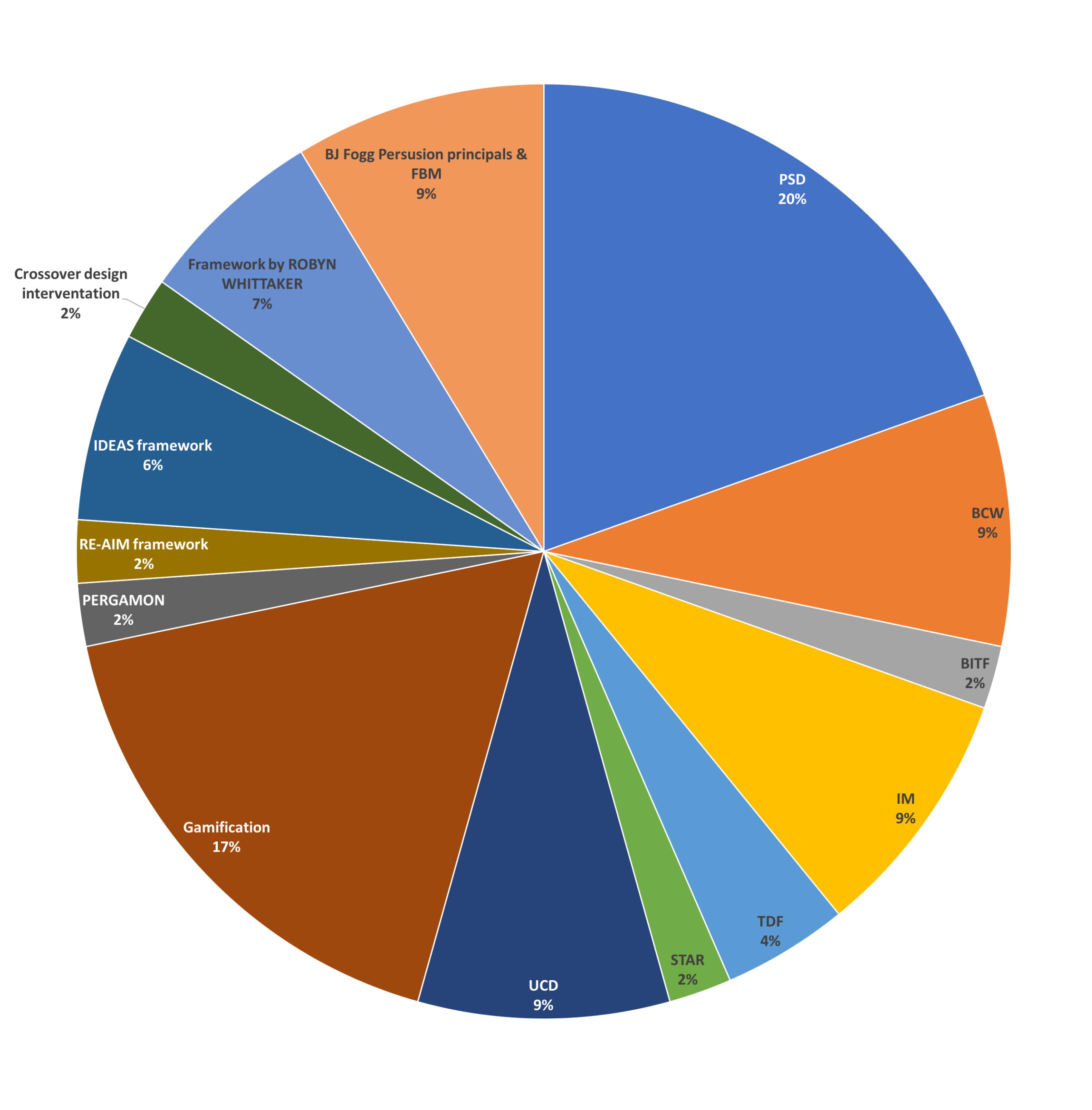

Table 1. The frequencies and references to all the adopted technologies.

Technology	Frequency	Reference
Mobile app	55.5% (62/118)	[1][2][3][4][5][6][7][8][9][10][11][12][13][14][15][16][17][18][19][20][21][22][23][24][25][26][27][28][29][30][31][32][33][34][35][36][37][38][39][40][41][42][43][44][45][46][47][48][49][50][51][52][53][54][55][56][57][58][59][60][61][62]
SMS	21.1% (25/118)	[1][2][63][64][65][66][67][68][69] [70] [71][72][73][74][75][69][76][77][78] [79][80] [81][82][83][84]
Web	19.4% (23/118)	[1][63] [5][85][86][87][88][89][27] [90][91][92][72] [38][46][93][94][95][96] [81][97][98][99]
Pedometers, sensors and wearable devices etc.	16.1% (19/118)	[63][10][100][101][22][23][31][102][41][73][48][52][53][78][97][60][103] [61] [62]
Others	09.3% (11/118)	Bluetooth Inhaler [12], haptic assisted physio-therapeutic system [26], virtual environment technology [104][105][106], telemonitoring [41], Interactive voice response technology [107][95], interactive posters [108], Augmented Bin [109] PDA [110]
Game	6.77% (8/118)	[85][111][112][113][114][25][115][116]
Desktop app	3.3% (4/118)	[12][27][32] [103]
Social networks	2.5% (3/118)	[109][117][60]

Table 2. Behavior change techniques included in the studies.

Technique	Usage count	Reference
Goal setting	42	[3][14][63][5][8][10][11][100][86][112][87][88][17][18][19][20][68][23][28][30][34] [70][35][90] [37][39][41][72][107][42] [73][45] [48][118][53][119][80][57][81] [98][103][99]
Self-monitoring	39	[3][63][85][11][13][100][15][16][112][18] [19][20][68][25][28][29][30][32][102][34] [70][35][92][71][40][41][72][107][44][49] [94][95][110][57][98][60][84][103][43]
Motivation	34	[2][63][4][6][9][10][111][14][67][25][27][31][69][70][104][91][92][38][39][42][73][44][109][105][48][115][76][54][80][58][60][61][99]

Feedback	33	[1][3][63][9][11][113][87][18][19][21][101][68][23][26][27][29][30][31][34][36][39][71][108][72][107][52][53][110][79][57][97][84][99]
Social support	21	[3][4][6][15][17][68][23][24][30][31][69][70][91][43] [94][117][54][79][57][98][60]
Self-belief/ Self-efficacy	14	[4][68][23][29][70][92][38][105][106][79][119][120][82][103]
Self-management	10	[5][7][65][22][43][46][47][50][51][58]
Reward/incentive	13	[85][8][111][87][114][18][21][23][33][49][79][99][62]
Self-regulation	8	[4][100][33][34][37][42][93][79]
Education/information	26	[63][64][4][5][85][9][66][12][113][87][114][18][19][68][23][24][33][35][38][43][45][75][119][80][57][59]
Tailoring/Personalization	3	[6][17][58]
Distraction	3	[6][68][54]
Discrepancy between current behavior and goal	2	[3][18]
Credible Source	2	[3][18][57]
Framing/reframing	2	[18][68]
Graded task	3	[3] [68][23]
Prompt/Cues	5	[18] [68][72][53][57]
Salience of Consequences	5	[3] [68][79][80][57]
Skill training	3	[4][119][58]
Positive Psychology technique	1	[13]
Reminder	2	[113][20][94][121][76][77][78]
Reinforcement	4	[113][52][121][77]
Social Comparison	2	[18][62]
empowerment	1	[61]

Reference

[1] C. Wright *et al.*, “Mobile Phone-Based Ecological Momentary Intervention to Reduce Young Adults’ Alcohol Use in the Event: A Three-Armed Randomized Controlled Trial,” *JMIR MHEALTH UHEALTH*, vol. 6, no. 7, Jul. 2018.

[2] S. Hors-Fraile *et al.*, “Tailoring motivational health messages for smoking cessation using an mHealth recommender system integrated with an electronic health record: a study protocol,” *BMC Public Health*, vol. 18, no. 1, p. 698, 2018.

[3] A. Direito *et al.*, “Using the Intervention Mapping and Behavioral Intervention Technology Frameworks: Development of an mHealth Intervention for Physical Activity and Sedentary Behavior Change,” *Heal. Educ. Behav.*, vol. 45, no. 3, pp. 331–348, 2018.

[4] J. E. M. van Agteren, S. Lawn, B. Bonevski, and B. J. Smith, “Kick. it: The development of an evidence-based smoking cessation smartphone app,” *Transl. Behav. Med.*, vol. 8, no. 2, pp. 243–267, 2018.

[5] D. Sarfati *et al.*, “BetaMe: impact of a comprehensive digital health programme on HbA1c and weight at 12 months for people with diabetes and pre-diabetes: study protocol for a randomised controlled trial,” *Trials*, vol. 19, no. 1, p. 161, 2018.

[6] N. B. Baskerville, L. L. Struik, and D. Dash, “Crush the Crave: Development and Formative Evaluation of a Smartphone App for Smoking Cessation,” *JMIR mHealth uHealth*, vol. 6, no. 3, 2018.

[7] G. Giunti, V. Mylonopoulou, and O. Rivera Romero, “More Stamina, a Gamified mHealth Solution for Persons with Multiple Sclerosis: Research Through Design,” *JMIR MHEALTH UHEALTH*, vol. 6, no. 3, Mar. 2018.

[8] E. V Korinek *et al.*, “Adaptive step goals and rewards: a longitudinal growth model of daily steps for a smartphone-based walking intervention,” *J. Behav. Med.*, vol. 41, no. 1, pp. 74–86, Feb. 2018.

[9] D. M. Kazemi, B. Borsari, M. J. Levine, K. A. Lamberson, and B. Dooley, “REMIT: Development of a mHealth theory-based intervention to decrease heavy episodic drinking among college students,” *Addict. Res. Theory*, vol. 26, no. 5, pp. 377–385, 2018.

[10] J. I. Recio-Rodriguez *et al.*, “EVIDENT 3 Study: A randomized, controlled clinical trial to reduce inactivity and caloric intake in sedentary and overweight or obese people using a smartphone application Study protocol,” *Medicine (Baltimore).*, vol. 97, no. 2, Jan. 2018.

[11] M. E. Whelan, A. P. Kingsnorth, M. W. Orme, L. B. Sherar, and D. W. Esliger, “Sensing interstitial glucose to nudge active lifestyles (SIGNAL): feasibility of combining novel self-monitoring technologies for persuasive behaviour change,” *BMJ Open*, vol. 7, no. 10, Oct. 2017.

[12] G. Humphris *et al.*, “Engagement in an e-Health Tool (ORION) predicts opioid-dependent patient likelihood of behavioural change,” *HEROIN Addict. Relat. Clin. Probl.*, vol. 19, no. 5, pp. 35–44, Oct. 2017.

[13] B. B. Hoeppner, S. S. Hoeppner, L. Kelly, M. Schick, and J. F. Kelly, “Smiling Instead of Smoking: Development of a Positive Psychology Smoking Cessation Smartphone App for Non-daily Smokers,” *Int. J. Behav. Med.*, vol. 24, no. 5, SI, pp. 683–693, Oct. 2017.

[14] J. Milward, P. Deluca, C. Drummond, R. Watson, J. Dunne, and A. Kimergard, “Usability Testing of the BRANCH Smartphone App Designed to Reduce Harmful Drinking in Young Adults,” *JMIR MHEALTH UHEALTH*, vol. 5, no. 8, Aug. 2017.

[15] B. Grossman *et al.*, “Application of Human Augmentics: A Persuasive Asthma Inhaler,” *J. Biomed. Inform.*, vol. 67, pp. 51–58, Mar. 2017.

[16] J. Freyne, J. Yin, E. Brindal, G. A. Hendrie, S. Berkovsky, and M. Noakes, “Push Notifications in Diet Apps: Influencing Engagement Times and Tasks,” *Int. J. Hum. Comput. Interact.*, vol. 33, no. 10, pp. 833–845, 2017.

[17] L. Oliveira, V. Mitchell, and A. May, “Reducing temporal tensions as a strategy to promote sustainable behaviours,” *Comput. Human Behav.*, vol. 62, pp. 303–315, Sep. 2016.

[18] S. A. Mummah, A. C. King, C. D. Gardner, and S. Sutton, “Iterative development of Vegethon: a theory-based mobile app intervention to increase vegetable consumption,” *Int. J. Behav. Nutr. Phys. Act.*, vol. 13, Aug. 2016.

[19] W. Van Lippevelde *et al.*, “Using a gamified monitoring app to change adolescents’ snack intake: the development of the REWARD app and evaluation design,” *BMC Public Health*, vol. 16, Aug. 2016.

[20] T. Batink *et al.*, “Acceptance and Commitment Therapy in Daily Life Training: A Feasibility Study of an mHealth Intervention,” *JMIR MHEALTH UHEALTH*, vol. 4,

no. 3, 2016.
[21] I. Bindoff *et al.*, “Quittr: The Design of a Video Game to Support Smoking Cessation,” *JMIR SERIOUS GAMES*, vol. 4, no. 2, 2016.
[22] D. Jones, N. Skrepnik, R. M. Toselli, and B. Leroy, “Incorporating Novel Mobile Health Technologies Into Management of Knee Osteoarthritis in Patients Treated With Intra-Articular Hyaluronic Acid: Rationale and Protocol of a Randomized Controlled Trial,” *JMIR Res. Protoc.*, vol. 5, no. 3, pp. 27–36, 2016.
[23] J. C. Rawstorn, N. Gant, A. Meads, I. Warren, and R. Maddison, “Remotely Delivered Exercise-Based Cardiac Rehabilitation: Design and Content Development of a Novel mHealth Platform,” *JMIR MHEALTH UHEALTH*, vol. 4, no. 2, pp. 430–445, 2016.
[24] B. K. White *et al.*, “Theory-Based Design and Development of a Socially Connected, Gamified Mobile App for Men About Breastfeeding (Milk Man),” *JMIR MHEALTH UHEALTH*, vol. 4, no. 2, pp. 566–580, 2016.
[25] E. J. Lyons *et al.*, “Testing the effects of narrative and play on physical activity among breast cancer survivors using mobile apps: study protocol for a randomized controlled trial,” *BMC Cancer*, vol. 16, Mar. 2016.
[26] V. Rajanna *et al.*, “KinoHaptics: An Automated, Wearable, Haptic Assisted, Physio-therapeutic System for Post-surgery Rehabilitation and Self-care,” *J. Med. Syst.*, vol. 40, no. 3, Mar. 2016.
[27] C. K. Martin, L. A. Gilmore, J. W. Apolzan, C. A. Myers, D. M. Thomas, and L. M. Redman, “Smartloss: A Personalized Mobile Health Intervention for Weight Management and Health Promotion,” *JMIR MHEALTH UHEALTH*, vol. 4, no. 1, pp. 223–234, 2016.
[28] E. Brindal, G. A. Hendrie, and J. Freyne, “Combining Persuasive Technology With Behavioral Theory to Support Weight Maintenance Through a Mobile Phone App: Protocol for the MotiMate App,” *JMIR Res. Protoc.*, vol. 5, no. 1, 2016.
[29] P. Giacobbi Jr., M. Hingle, T. Johnson, J. K. Cunningham, J. Armin, and J. S. Gordon, “See Me Smoke-Free: Protocol for a Research Study to Develop and Test the Feasibility of an mHealth App for Women to Address Smoking, Diet, and Physical Activity,” *JMIR Res. Protoc.*, vol. 5, no. 1, 2016.
[30] M. D. Hingle *et al.*, “The EPIC Kids Study: a randomized family-focused YMCA-based intervention to prevent type 2 diabetes in at-risk youth,” *BMC Public Health*, vol. 15, Dec. 2015.
[31] S. S. Martin *et al.*, “mActive: A Randomized Clinical Trial of an Automated mHealth Intervention for Physical Activity Promotion,” *J. Am. Heart Assoc.*, vol. 4, no. 11, Nov. 2015.
[32] L. Cadmus-Bertram, B. H. Marcus, R. E. Patterson, B. A. Parker, and B. L. Morey, “Use of the Fitbit to Measure Adherence to a Physical Activity Intervention Among Overweight or Obese, Postmenopausal Women: Self-Monitoring Trajectory During 16 Weeks,” *JMIR MHEALTH UHEALTH*, vol. 3, no. 4, pp. 82–88, 2015.
[33] M. Hassandra *et al.*, “Effectiveness of a Mobile Phone App for Adults That Uses Physical Activity as a Tool to Manage Cigarette Craving After Smoking Cessation: A Study Protocol for a Randomized Controlled Trial,” *JMIR Res. Protoc.*, vol. 4, no. 4, 2015.
[34] A. Direito, Y. Jiang, R. Whittaker, and R. Maddison, “Smartphone apps to improve fitness and increase physical activity among young people: protocol of the Apps for IMproving FITness (AIMFIT) randomized controlled trial,” *BMC Public Health*, vol. 15, Jul. 2015.
[35] K. E. Curtis, S. Lahiri, and K. E. Brown, “Targeting Parents for Childhood Weight Management: Development of a Theory-Driven and User-Centered Healthy Eating

App," *JMIR MHEALTH UHEALTH*, vol. 3, no. 2, 2015.
[36] M. Rabbi, A. Pfammatter, M. Zhang, B. Spring, and T. Choudhury, "Automated Personalized Feedback for Physical Activity and Dietary Behavior Change With Mobile Phones: A Randomized Controlled Trial on Adults," *JMIR MHEALTH UHEALTH*, vol. 3, no. 2, 2015.
[37] C. A. Pellegrini *et al.*, "Design and protocol of a randomized multiple behavior change trial: Make Better Choices 2 (MBC2)," *Contemp. Clin. Trials*, vol. 41, pp. 85–92, Mar. 2015.
[38] D. Modi *et al.*, "Development and formative evaluation of an innovative mHealth intervention for improving coverage of community-based maternal, newborn and child health services in rural areas of India," *Glob. Health Action*, vol. 8, pp. 1–14, 2015.
[39] J. Barnett, M. Harricharan, D. Fletcher, B. Gilchrist, and J. Coughlan, "myPace: An Integrative Health Platform for Supporting Weight Loss and Maintenance Behaviors," *IEEE J. Biomed. Heal. INFORMATICS*, vol. 19, no. 1, pp. 109–116, Jan. 2015.
[40] M. Klein, N. Mogles, and A. van Wissen, "Intelligent mobile support for therapy adherence and behavior change," *J. Biomed. Inform.*, vol. 51, pp. 137–151, Oct. 2014.
[41] H. Holmen *et al.*, "A Mobile Health Intervention for Self-Management and Lifestyle Change for Persons With Type 2 Diabetes, Part 2: One-Year Results From the Norwegian Randomized Controlled Trial RENEWING HEALTH," *JMIR MHEALTH UHEALTH*, vol. 2, no. 4, 2014.
[42] S. McMahon, M. Vankipuram, E. B. Hekler, and J. Fleury, "Design and evaluation of theory-informed technology to augment a wellness motivation intervention," *Transl. Behav. Med.*, vol. 4, no. 1, pp. 95–107, Mar. 2014.
[43] S. Nundy, J. J. Dick, C.-H. Chou, R. S. Nocon, M. H. Chin, and M. E. Peek, "Mobile Phone Diabetes Project Led To Improved Glycemic Control And Net Savings For Chicago Plan Participants," *Health Aff.*, vol. 33, no. 2, pp. 265–272, Feb. 2014.
[44] A. Khalil and S. Abdallah, "Harnessing social dynamics through persuasive technology to promote healthier lifestyle," *Comput. Human Behav.*, vol. 29, no. 6, pp. 2674–2681, Nov. 2013.
[45] R. Orji, J. Vassileva, and R. L. Mandryk, "LunchTime: a slow-casual game for long-term dietary behavior change," *Pers. Ubiquitous Comput.*, vol. 17, no. 6, pp. 1211–1221, 2013.
[46] J. Mintz and M. Aagaard, "The application of persuasive technology to educational settings," *ETR&D-EDUCATIONAL Technol. Res. Dev.*, vol. 60, no. 3, pp. 483–499, Jun. 2012.
[47] L. Ribu *et al.*, "Low-intensity self-management intervention for persons with type 2 diabetes using a mobile phone-based diabetes diary, with and without health counseling and motivational interviewing: Protocol for a randomized controlled trial," *J. Med. Internet Res.*, vol. 15, no. 8, 2013.
[48] S. Consolvo, J. A. Landay, and D. W. McDonald, "Designing for behavior change in everyday life," *Computer (Long. Beach. Calif).*, vol. 42, no. 6, pp. 86–89, 2009.
[49] J. B. Bricker *et al.*, "Randomized, controlled pilot trial of a smartphone app for smoking cessation using acceptance and commitment therapy," *Drug Alcohol Depend.*, vol. 143, no. 1, pp. 87–94, 2014.
[50] O. Duff *et al.*, "MedFit app, a behavior-changing, theoretically informed mobile app for patient self-management of cardiovascular disease: User-centered development," *J. Med. Internet Res.*, vol. 20, no. 4, 2018.
[51] S. Mummah, T. N. Robinson, M. Mathur, S. Farzinkhou, S. Sutton, and C. D. Gardner, "Effect of a mobile app intervention on vegetable consumption in overweight adults: A randomized controlled trial," *Int. J. Behav. Nutr. Phys. Act.*, vol. 14, no. 1, 2017.

[52] R. Cattivelli *et al.*, "ACTonHEALTH study protocol: Promoting psychological flexibility with activity tracker and mHealth tools to foster healthful lifestyle for obesity and other chronic health conditions," *Trials*, vol. 19, no. 1, 2018.
[53] J. Graham Thomas and D. S. Bond, "Behavioral response to a just-in-time adaptive intervention (JITAI) to reduce sedentary behavior in obese adults: Implications for JITAI optimization," *Heal. Psychol.*, vol. 34, pp. 1261–1267, 2015.
[54] K. Ghorai, S. Jahan, P. Ray, and M. Chylinski, "MHealth for behaviour change: Role of a smartphonebased multi-intervention service for hypertension and diabetes in Bangladesh," *Int. J. Biomed. Eng. Technol.*, vol. 16, no. 2, pp. 135–155, 2014.
[55] Z. Yang, X. Kong, J. Sun, and Y. Zhang, "Switching to green lifestyles: Behavior change of ant forest users," *Int. J. Environ. Res. Public Health*, vol. 15, no. 9, 2018.
[56] M. H. M. Salim, N. M. Ali, and S. A. M. Noah, "Mobile application on healthy diet for elderly based on persuasive design," *Int. J. Adv. Sci. Eng. Inf. Technol.*, vol. 7, no. 1, pp. 222–227, 2017.
[57] N. Chiang, M. Guo, K. R. Amico, L. Atkins, and R. T. Lester, "Interactive two-way mhealth interventions for improving medication adherence: An evaluation using the behaviour change wheel framework," *JMIR mHealth uHealth*, vol. 6, no. 4, 2018.
[58] K. L. Fortuna *et al.*, "Certified Peer Specialists and Older Adults with Serious Mental Illness' Perspectives of the Impact of a Peer-Delivered and Technology-Supported Self-Management Intervention," *J. Nerv. Ment. Dis.*, vol. 206, no. 11, pp. 875–881, 2018.
[59] A. van Drongelen, C. R. L. Boot, H. Hlobil, J. W. R. Twisk, T. Smid, and A. J. van der Beek, "Evaluation of an mHealth intervention aiming to improve health-related behavior and sleep and reduce fatigue among airline pilots," *Scand. J. Work. Environ. Heal.*, vol. 40, no. 6, pp. 557–568, 2014.
[60] K. A. Aschbrenner, J. A. Naslund, M. Shevenell, K. T. Mueser, and S. J. Bartels, "Feasibility of Behavioral Weight Loss Treatment Enhanced with Peer Support and Mobile Health Technology for Individuals with Serious Mental Illness," *Psychiatr. Q.*, vol. 87, no. 3, pp. 401–415, 2016.
[61] D. H. Griauzde, J. T. Kullgren, B. Liestenfeltz, C. Richardson, and M. Heisler, "A mobile phone-based program to promote healthy behaviors among adults with prediabetes: Study protocol for a pilot randomized controlled trial," *Pilot Feasibility Stud.*, vol. 4, no. 1, 2018.
[62] K. van Turnhout, J. Jeurens, M. Verhey, P. Wientjes, and R. Bakker, "The healthy elderly: Case studies in persuasive design," *Interact. Des. Archit.*, vol. 23, no. 1, pp. 160–172, 2014.
[63] M. Marthick, H. M. Dhillon, J. A. Alison, B. S. Cheema, and T. Shaw, "Development of a Web Portal for Physical Activity and Symptom Tracking in Oncology Patients: Protocol for a Prospective Cohort Study," *JMIR Res. Protoc.*, vol. 7, no. 5, 2018.
[64] C. Brody, S. Tuot, P. Chhoun, D. Swendenman, K. C. Kaplan, and S. Yi, "Mobile Link--a theory-based messaging intervention for improving sexual and reproductive health of female entertainment workers in Cambodia: study protocol of a randomized controlled trial," *Trials*, vol. 19, no. 1, p. 235, 2018.
[65] R. Maddison *et al.*, "Text4Heart II - improving medication adherence in people with heart disease: a study protocol for a randomized controlled trial," *Trials*, vol. 19, Jan. 2018.
[66] H. Lee, R. Ghebre, C. Le, Y. J. Jang, M. Sharratt, and D. Yee, "Mobile Phone Multilevel and Multimedia Messaging Intervention for Breast Cancer Screening: Pilot Randomized Controlled Trial," *JMIR MHEALTH UHEALTH*, vol. 5, no. 11, Nov. 2017.

[67] Y. Liao *et al.*, “The efficacy of mobile phone-based text message interventions ('Happy Quit’) for smoking cessation in China,” *BMC Public Health*, vol. 16, Aug. 2016.
[68] R. Dobson *et al.*, “Text message-based diabetes self-management support (SMS4BG): study protocol for a randomised controlled trial,” *Trials*, vol. 17, Apr. 2016.
[69] N. Leon, R. Surender, K. Bobrow, J. Muller, and A. Farmer, “Improving treatment adherence for blood pressure lowering via mobile phone SMS-messages in South Africa: a qualitative evaluation of the SMS-text Adherence SuppoRt (StAR) trial,” *BMC Fam. Pract.*, vol. 16, Jul. 2015.
[70] A. F. Yan *et al.*, “mHealth Text Messaging for Physical Activity Promotion in College Students: A Formative Participatory Approach,” *Am. J. Health Behav.*, vol. 39, no. 3, pp. 395–408, May 2015.
[71] E. J. Ambeba *et al.*, “The Use of mHealth to Deliver Tailored Messages Reduces Reported Energy and Fat Intake,” *J. Cardiovasc. Nurs.*, vol. 30, no. 1, pp. 35–43, 2015.
[72] W. Waterlander *et al.*, “Development of an Evidence-Based mHealth Weight Management Program Using a Formative Research Process,” *JMIR MHEALTH UHEALTH*, vol. 2, no. 3, 2014.
[73] M. A. Adams, J. F. Sallis, G. J. Norman, M. F. Hovell, E. B. Hekler, and E. Perata, “An Adaptive Physical Activity Intervention for Overweight Adults: A Randomized Controlled Trial,” *PLoS One*, vol. 8, no. 12, Dec. 2013.
[74] E. Burn, A. L. Marshall, Y. D. Miller, A. G. Barnett, B. S. Fjeldsoe, and N. Graves, “The cost-effectiveness of the MobileMums intervention to increase physical activity among mothers with young children: a Markov model informed by a randomised controlled trial,” *BMJ Open*, vol. 5, no. 4, p. e007226, 2015.
[75] D. Johnson *et al.*, “A randomized controlled trial of the impact of a family planning mHealth service on knowledge and use of contraception,” *Contraception*, vol. 95, no. 1, pp. 90–97, 2017.
[76] A. M. Müller, S. Khoo, and T. Morris, “Text Messaging for Exercise Promotion in Older Adults From an Upper-Middle-Income Country: Randomized Controlled Trial,” *J. Med. Internet Res.*, vol. 18, no. 1, p. e5, 2016.
[77] D. V Wagner, S. A. Barry, M. Stoeckel, L. Teplitsky, and M. A. Harris, “NICH at Its Best for Diabetes at Its Worst: Texting Teens and Their Caregivers for Better Outcomes,” *J. Diabetes Sci. Technol.*, vol. 11, no. 3, pp. 468–475, 2017.
[78] M. T. J. Heino, K. Knittle, A. Haukkala, T. Vasankari, and N. Hankonen, “Simple and rationale-providing SMS reminders to promote accelerometer use: A within-trial randomised trial comparing persuasive messages,” *BMC Public Health*, vol. 18, no. 1, 2018.
[79] M. Lauti, M. Kularatna, A. Pillai, A. G. Hill, and A. D. MacCormick, “A Randomised Trial of Text Message Support for Reducing Weight Regain Following Sleeve Gastrectomy,” *Obes. Surg.*, vol. 28, no. 8, pp. 2178–2186, 2018.
[80] K. L. Brookie, L. A. Mainvil, A. C. Carr, M. C. M. Vissers, and T. S. Conner, “The development and effectiveness of an ecological momentary intervention to increase daily fruit and vegetable consumption in low-consuming young adults,” *Appetite*, vol. 108, pp. 32–41, 2017.
[81] L. P. Dale *et al.*, “Acceptability of a Mobile Health Exercise-Based Cardiac Rehabilitation Intervention A RANDOMIZED TRIAL,” *J. Cardiopulm. Rehabil. Prev.*, vol. 35, no. 5, pp. 312–319, 2015.
[82] L. P. Dale *et al.*, “Cardiovascular disease self-management: Pilot testing of an mhealth healthy eating program,” *J. Pers. Med.*, vol. 4, no. 1, pp. 88–101, 2014.

[83] B. McBride, J. D. O'Neil, T. T. Hue, R. Eni, C. Vu Nguyen, and L. T. Nguyen, "Improving health equity for ethnic minority women in Thai Nguyen, Vietnam: Qualitative results from an mHealth intervention targeting maternal and infant health service access," *J. Public Heal. (United Kingdom)*, vol. 40, pp. II32–II41, 2018.
[84] T. Irizarry *et al.*, "Development and Preliminary Feasibility of an Automated Hypertension Self-Management System," *Am. J. Med.*, vol. 131, no. 9, pp. 1125.e1-1125.e8, 2018.
[85] R. Klaassen, K. Bul, G. J. van der Burg, P. M. Kato, P. Di Bitonto, and others, "Design and Evaluation of a Pervasive Coaching and Gamification Platform for Young Diabetes Patients," *Sensors*, vol. 18, no. 2, p. 402, 2018.
[86] C. E. Short *et al.*, "How do different delivery schedules of tailored web-based physical activity advice for breast cancer survivors influence intervention use and efficacy?," *J. CANCER Surviv.*, vol. 11, no. 1, pp. 80–91, Feb. 2017.
[87] L. Neubeck *et al.*, "Development of an integrated e-health tool for people with, or at high risk of, cardiovascular disease: The Consumer Navigation of Electronic Cardiovascular Tools (CONNECT) web application," *Int. J. Med. Inform.*, vol. 96, pp. 24–37, Dec. 2016.
[88] H. M. Mohadis, N. Mohamad Ali, and A. F. Smeaton, "Designing a persuasive physical activity application for older workers: understanding end-user perceptions," *Behav. Inf. Technol.*, vol. 35, no. 12, pp. 1102–1114, Dec. 2016.
[89] S. Lin, "Reducing students' carbon footprints using personal carbon footprint management system based on environmental behavioural theory and persuasive technology," *Environ. Educ. Res.*, vol. 22, no. 5, pp. 658–682, Jul. 2016.
[90] S. Mantwill, M. Fiordelli, R. Ludolph, and P. J. Schulz, "EMPOWER-support of patient empowerment by an intelligent self-management pathway for patients: study protocol," *BMC Med. Inform. Decis. Mak.*, vol. 15, Mar. 2015.
[91] N. Weymann, J. Dirmaier, A. von Wolff, L. Kriston, and M. Haerter, "Effectiveness of a Web-Based Tailored Interactive Health Communication Application for Patients With Type 2 Diabetes or Chronic Low Back Pain: Randomized Controlled Trial," *J. Med. INTERNET Res.*, vol. 17, no. 3, Mar. 2015.
[92] M. Moreau, M.-P. Gagnon, and F. Boudreau, "Development of a Fully Automated, Web-Based, Tailored Intervention Promoting Regular Physical Activity Among Insufficiently Active Adults With Type 2 Diabetes: Integrating the I-Change Model, Self-Determination Theory, and Motivational Interviewing Compon," *JMIR Res. Protoc.*, vol. 4, no. 1, 2015.
[93] J. Plaete, G. Crombez, C. Van Der Mispel, M. Verloigne, V. Van Stappen, and I. De Bourdeaudhuij, "Effect of the Web-based intervention MyPlan 1.0 on self-reported fruit and vegetable intake in adults who visit general practice: A quasi-experimental trial," *J. Med. Internet Res.*, vol. 18, no. 2, 2016.
[94] P. Karppinen *et al.*, "Opportunities and challenges of behavior change support systems for enhancing habit formation: A qualitative study," *J. Biomed. Inform.*, vol. 84, pp. 82–92, 2018.
[95] K. Y. Wolin *et al.*, "Engagement with eHealth self-monitoring in a primary care-based weight management intervention," *PLoS One*, vol. 10, no. 10, 2015.
[96] C. Valk *et al.*, "Towards personalized persuasive strategies for active ageing," *Gerontechnology*, vol. 16, no. 3, pp. 160–172, 2017.
[97] V. Sundramoorthy, G. Cooper, N. Linge, and Q. Liu, "Domesticating energy-monitoring systems: Challenges and design concerns," *IEEE Pervasive Comput.*, vol. 10, no. 1, pp. 20–27, 2011.
[98] A.-M. Teeriniemi *et al.*, "A randomized clinical trial of the effectiveness of a Web-

based health behaviour change support system and group lifestyle counselling on body weight loss in overweight and obese subjects: 2-year outcomes," *J. Intern. Med.*, vol. 284, no. 5, pp. 534–545, 2018.

[99] H. Kimura and T. Nakajima, "Designing persuasive applications to motivate sustainable behavior in collectivist cultures," *PsychNology J.*, vol. 9, no. 1, pp. 7–28, 2011.

[100] J. Gaudet, F. Gallant, and M. Belanger, "A Bit of Fit: Minimalist Intervention in Adolescents Based on a Physical Activity Tracker," *JMIR MHEALTH UHEALTH*, vol. 5, no. 7, Jul. 2017.

[101] N. M. Oreskovic, R. Fletcher, M. Sharifi, J. D. Knutsen, A. Chilingirian, and E. M. Taveras, "Design and rationale of the STRIVE trial to improve cardiometabolic health among children and families," *Contemp. Clin. Trials*, vol. 49, pp. 149–154, Jul. 2016.

[102] L. A. Cadmus-Bertram, B. H. Marcus, R. E. Patterson, B. A. Parker, and B. L. Morey, "Randomized Trial of a Fitbit-Based Physical Activity Intervention for Women," *Am. J. Prev. Med.*, vol. 49, no. 3, pp. 414–418, Sep. 2015.

[103] S. Mawson, N. Nasr, J. Parker, H. Zheng, R. Davies, and G. Mountain, "Developing a personalised self-management system for post stroke rehabilitation; utilising a user-centred design methodology," *Disabil. Rehabil. Assist. Technol.*, vol. 9, no. 6, pp. 521–528, 2014.

[104] L. C. Brewer, B. Kaihoi, K. K. Zarling, R. W. Squires, R. Thomas, and S. Kopecky, "The Use of Virtual World-Based Cardiac Rehabilitation to Encourage Healthy Lifestyle Choices Among Cardiac Patients: Intervention Development and Pilot Study Protocol," *JMIR Res. Protoc.*, vol. 4, no. 2, 2015.

[105] E. A. Plant, A. L. Baylor, C. E. Doerr, and R. B. Rosenberg-Kima, "Changing middle-school students' attitudes and performance regarding engineering with computer-based social models," *Comput. Educ.*, vol. 53, no. 2, pp. 209–215, Sep. 2009.

[106] J. Fox and J. N. Bailenson, "Virtual Self-Modeling: The Effects of Vicarious Reinforcement and Identification on Exercise Behaviors," *MEDIA Psychol.*, vol. 12, no. 1, pp. 1–25, 2009.

[107] D. M. Steinberg *et al.*, "Adherence to Self-Monitoring via Interactive Voice Response Technology in an eHealth Intervention Targeting Weight Gain Prevention Among Black Women: Randomized Controlled Trial," *J. Med. INTERNET Res.*, vol. 16, no. 4, pp. 105–116, Apr. 2014.

[108] M. M. Agha-Hossein *et al.*, "Providing persuasive feedback through interactive posters to motivate energy-saving behaviours," *Intell. Build. Int.*, vol. 7, no. 1, pp. 16–35, 2015.

[109] R. Comber and A. Thieme, "Designing beyond habit: opening space for improved recycling and food waste behaviors through processes of persuasion, social influence and aversive affect," *Pers. UBIQUITOUS Comput.*, vol. 17, no. 6, SI, pp. 1197–1210, Aug. 2013.

[110] L. E. Burke *et al.*, "The SMARTER pilot study: Testing feasibility of real-time feedback for dietary self-monitoring," *Prev. Med. Reports*, vol. 6, pp. 278–285, 2017.

[111] R. Orji, R. L. Mandryk, and J. Vassileva, "Improving the Efficacy of Games for Change Using Personalization Models," *ACM Trans. Comput. Interact.*, vol. 24, no. 5, Nov. 2017.

[112] A. A. Lyles, A. Amresh, J. Huberty, M. Todd, and R. E. Lee, "A Mobile, Avatar-Based App for Improving Body Perceptions Among Adolescents: A Pilot Test," *JMIR SERIOUS GAMES*, vol. 5, no. 1, 2017.

[113] D. A. Kuipers *et al.*, "iLift: A health behavior change support system for lifting and transfer techniques to prevent lower-back injuries in healthcare," *Int. J. Med. Inform.*,

vol. 96, pp. 11–23, Dec. 2016.

[114] B. W. Lukhele *et al.*, “Efficacy of Mobile Serious Games in Increasing HIV Risk Perception in Swaziland: A Randomized Control Trial (SGprev Trial) Research Protocol,” *JMIR Res. Protoc.*, vol. 5, no. 4, 2016.

[115] M.-H. Tsai, Y.-L. Chang, C. Kao, and S.-C. Kang, “The effectiveness of a flood protection computer game for disaster education,” *Vis. Eng.*, vol. 3, no. 1, 2015.

[116] K. Winskell *et al.*, “A smartphone game-based intervention (Tumaini) to prevent HIV among young Africans: Pilot randomized controlled trial,” *J. Med. Internet Res.*, vol. 20, no. 8, 2018.

[117] S. J. Kim, L. A. Marsch, M. F. Brunette, and J. Dallery, “Harnessing Facebook for Smoking Reduction and Cessation Interventions: Facebook User Engagement and Social Support Predict Smoking Reduction,” *J. Med. Internet Res.*, vol. 19, no. 5, p. e168, 2017.

[118] S. Verdaguer, K. F. Mateo, K. Wyka, T. A. Dennis-Tiwary, and M. M. Leung, “A web-based interactive tool to reduce childhood obesity risk in urban minority youth: Usability testing study,” *J. Med. Internet Res.*, vol. 20, no. 11, 2018.

[119] K. Winskell *et al.*, “A smartphone game-based intervention (Tumaini) to prevent HIV among young africans: Pilot randomized controlled trial,” *JMIR mHealth uHealth*, vol. 6, no. 8, 2018.

[120] L. Pfaeffli Dale *et al.*, “Acceptability of a mobile health exercise-based cardiac rehabilitation intervention: A randomized trial,” *J. Cardiopulm. Rehabil. Prev.*, vol. 35, no. 5, pp. 312–319, 2015.

[121] N. Leon, R. Surender, K. Bobrow, J. Muller, and A. Farmer, “Improving treatment adherence for blood pressure lowering via mobile phone SMS-messages in South Africa: A qualitative evaluation of the SMS-text Adherence SuppoRt (StAR) trial Service organization, utilization, and delivery of care,” *BMC Fam. Pract.*, vol. 16, no. 1, 2015.
